# Constant Light Versus Intermittent Constant Light/Recovery Regimen: Differential Effects on Pineal Ultrastructure, Clock Gene Protein Abundance, and Systemic Metabolism in Rats

**DOI:** 10.3390/ijms27198817

**Published:** 2026-10-02

**Authors:** Anna I. Anurkina, Maria A. Kozlova, Valery P. Chernikov, Nikita S. Gladyshev, Larisa E. Zavalishina, Boris N. Kulbitskiy, David A. Areshidze

**Affiliations:** 1 Petrovsky National Research Centre of Surgery, 119435 Moscow, Russia; 2 Department of Pathological Anatomy, Russian Medical Academy of Continuous Professional Education, 125993 Moscow, Russia

**Keywords:** pineal gland, melatonin, circadian disruption, constant light, intermittent constant light, ultrastructure, clock genes, oxidative stress, lysosomes, metabolic syndrome

## Abstract

Disruption of the natural light regimen is considered a key pathogenetic factor in the development of desynchronosis and associated diseases. The pineal gland, as a neuroendocrine organ, is a central component of the circadian system and the primary target of the pathogenic effects of disrupted lighting. However, it remains unclear whether the mechanisms of pineal damage differ between different types of light stress. In this study, 120 male Wistar rats underwent a comprehensive analysis of the morphofunctional state of the pineal gland under two lighting regimens: constant lighting and intermittent constant light. Using light and electron microscopy, immunohistochemistry, ELISA, and cosinor analysis, it was established that the two regimens induce qualitatively different types of light stress. The obtained data indicate that constant lighting induces a degenerative phenotype with loss of rhythmic organization, whereas the shift regimen produces a phenotype of chronic re-adaptation with hyperactivation of the lysosomal system, paradoxical overexpression of clock genes, and pronounced metabolic disturbances. The identified differential markers may serve as a basis for preclinical evaluation of the efficacy of chronobiotic therapy and for the development of prevention strategies for disorders associated with shift work and light pollution.

## 1. Introduction

Modern civilization is characterized by an unprecedented level of light pollution. This includes everything from the widespread use of screens that emit blue-spectrum light and frequent transcontinental flights to the large-scale artificial lighting of the night sky in urbanized areas and the widespread adoption of round-the-clock and shift work schedules. This has led to the creation of fundamentally new environmental conditions to which biological species, including humans, are not evolutionarily adapted [1,2]. The fundamental basis of vital activity for the vast majority of organisms is circadian rhythms—endogenous, genetically determined oscillations of physiological and biochemical processes with a period of 24 ± 4 h [3,4]. Disruption of the light regime causes desynchronosis, i.e., a mismatch between endogenous rhythms and external time signals (primarily the light–dark cycle) and among themselves, and is regarded as one of the leading pathogenetic factors in the development of metabolic syndrome, obesity, type 2 diabetes mellitus, cardiovascular diseases, affective disorders, neurodegenerative diseases, and malignancies [5,6,7,8,9].

The central neuroendocrine organ in the system regulating circadian rhythms is the pineal gland [10]. This small organ synthesizes and secretes the hormone melatonin at night [11,12,13,14,15]. Even a brief exposure to a light source can suppress melatonin secretion at this time, triggering “circadian rhythm disorders” and the development of desynchronosis [16,17,18].

Beyond its role in daily circadian regulation, the pineal gland and its melatonin signal are also critically involved in photoperiodic responses—the transduction of seasonal changes in day length into physiological adaptations. In seasonally breeding mammals such as hamsters and sheep, the duration of nocturnal melatonin secretion provides a hormonal code for day length, regulating reproductive function, coat color changes, and metabolic adaptations to seasonal changes [19,20]. This photoperiodic function, while not the focus of the present study, underscores the evolutionary significance of the pineal gland as a transducer of environmental light information across multiple timescales.

At the molecular level, cellular circadian rhythmicity is provided by a transcription-translation feedback loop formed by “clock” genes. Heterodimeric activators CLOCK and BMAL1 induce the expression of repressor genes *Per* (Period 1, 2, 3) and *Cry* (Cryptochrome 1, 2). The PER and CRY proteins, accumulating in the cytoplasm, translocate into the nucleus and suppress CLOCK/BMAL1 activity, completing the cycle in approximately 24 h [21,22]. The pineal gland, although lacking its own autonomous oscillator activity, expresses a full set of clock genes, the expression of which is under strict control of the suprachiasmatic nuclei via the polysynaptic sympathetic pathway [23,24].

The morphology of the mammalian pineal gland has been described in detail in a number of classic works. The parenchyma of the gland mainly consists of two types of cells: light pinealocytes (type I)—large secretory cells with extensive cytoplasm rich in organelles, and dark pinealocytes (type II)—smaller cells with dense cytoplasm, considered to be low-productive or degenerating forms [25,26,27]. Pinealocytes are characterized by the presence of secretory granules, well-developed Golgi complexes, endoplasmic reticula, numerous mitochondria, and specific structures such as synaptic ribbons [28,29].

The effect of light on pineal function is a cornerstone of chronobiology. It has been established that even brief light exposure during the night, especially in the blue part of the spectrum, leads, through activation of the melanopsin system, to the suppression of sympathetic stimulation of the pineal gland, a sharp drop in arylalkylamine N-acetyltransferase (AANAT) activity, and an almost complete cessation of melatonin secretion [30,31]. Experimental studies in rodents show that prolonged constant lighting or inversion of the light–dark regimen leads to functional and structural changes in the pineal gland: a decrease in the size of pinealocyte nuclei and cytoplasm, reduction in the endoplasmic reticulum and Golgi complex, and a decrease in the number of secretory granules and mitochondria [32,33,34].

Previous studies have demonstrated that constant light and disrupted light–dark cycles alter the structure and cellular composition of the pineal gland. However, most of these investigations focused on a single level of analysis (e.g., either ultrastructure or gene expression) or examined only extreme photic conditions such as complete darkness or constant light. A significant gap in the literature remains regarding whether different types of light regimen disruption—particularly intermittent patterns that more closely model real-world scenarios such as irregular shift schedules or fragmented night work—produce qualitatively different phenotypes of pineal damage rather than merely quantitative intensification of the same effects. The question of whether the mechanisms of pineal damage differ between direct photic suppression and circadian desynchronization has yet to be systematically addressed in a single comprehensive study.

The novelty of our study lies in three key aspects:

The first, to our knowledge, this is the first study to demonstrate that constant lighting (direct photic suppression) and an intermittent constant light/recovery regimen (circadian desynchronization) induce qualitatively different phenotypes of pinealocyte damage, rather than merely a quantitative intensification of the same effects. While previous studies have examined pineal responses to either constant light or altered photoperiods in isolation, none have directly compared these two distinct types of light stress within a single experimental framework using identical analytical methods. Our results reveal that constant lighting produces a degenerative phenotype characterized by severe atrophy, secretory apparatus suppression, and complete loss of rhythmicity, whereas the intermittent regimen induces a phenotype of chronic re-adaptation with paradoxical preservation of melatonin rhythm, hyperactivation of the lysosomal system, and profound clock gene dysregulation.

The second, we provide a truly multi-level analysis that simultaneously integrates:

- Ultrastructural parameters (mitochondrial cristae number and morphology, Golgi complex integrity, lysosomal fraction, autophagosome abundance);
- Molecular markers (CLOCK and PER2 protein immunoreactivity with circadian dynamics);
- Functional outputs (daily melatonin secretion profiles and systemic metabolic parameters including lipid and carbohydrate metabolism);
- Chronobiological metrics (cosinor analysis of acrophases, amplitudes, and rhythmicity across 24 parameters).

This comprehensive approach allows, for the first time, the identification of specific, organelle-level markers that discriminate between the two types of light stress, and reveals how structural damage at the subcellular level translates into systemic metabolic disturbances.

The third, we employ advanced multivariate statistical methods (PCA, PERMANOVA, regularized logistic regression with elastic net) that achieve 100% classification accuracy between the three experimental groups. This demonstrates that the set of 24 studied parameters constitutes a robust and reliable diagnostic tool for differentiating types of light stress, with more than 70% of the variance in ultrastructural and molecular parameters attributable to the lighting regimen. The quantitative effect size analysis (η^2^ and Cohen’s d) further allows ranking of the most sensitive markers for each type of damage, providing a basis for future preclinical screening of chronobiotic interventions.

The aim of this study is to investigate the effects of various lighting regimens on the morphofunctional state of the pineal gland and the circadian homeostasis of Wistar rats, with an emphasis on identifying differential markers of the direct damaging and desynchronizing effects of light.

## 2. Results

### 2.1. Body Weight

**Figure 1:**
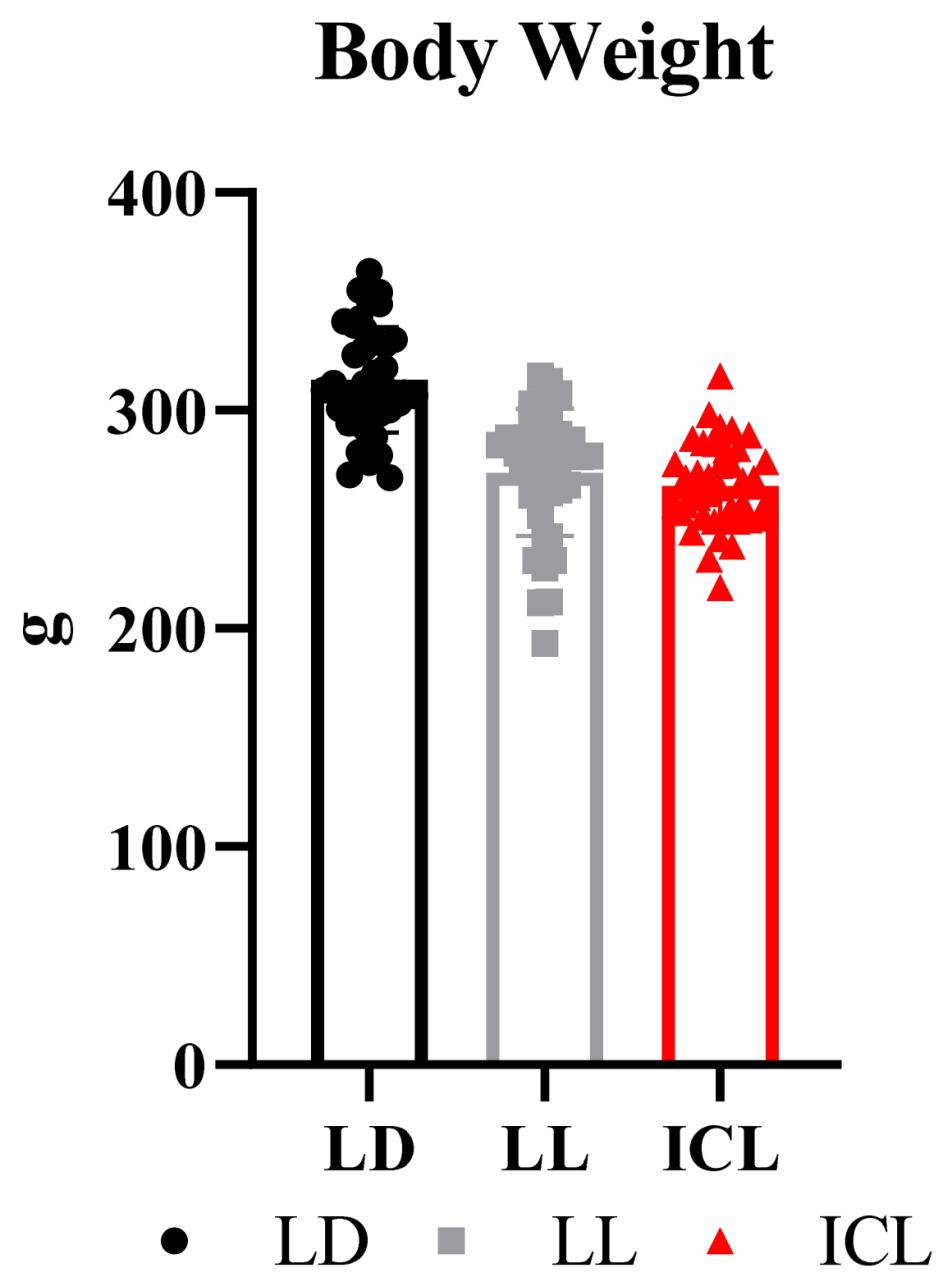
Body weight of the rats.

By the end of the 21-day experiment, the body weight of animals in the control group was 314.40 ± 20.74 g. In rats exposed to constant lighting (LL), body weight significantly decreased to 272.30 ± 25.94 g, and in animals on the intermittent constant light/recovery regimen (ICL), 264.71 ± 18.83 g. The differences between the experimental groups were also significant. The most pronounced suppression of weight gain was observed in ICL (a 15.8% reduction relative to the control versus 13.4% in LL) (Figure 1). Initial body weight did not differ between groups (*p* = 0.87).

### 2.2. Light-Optical Morphology and Cellularity of the Pineal Gland

**Figure 2:**
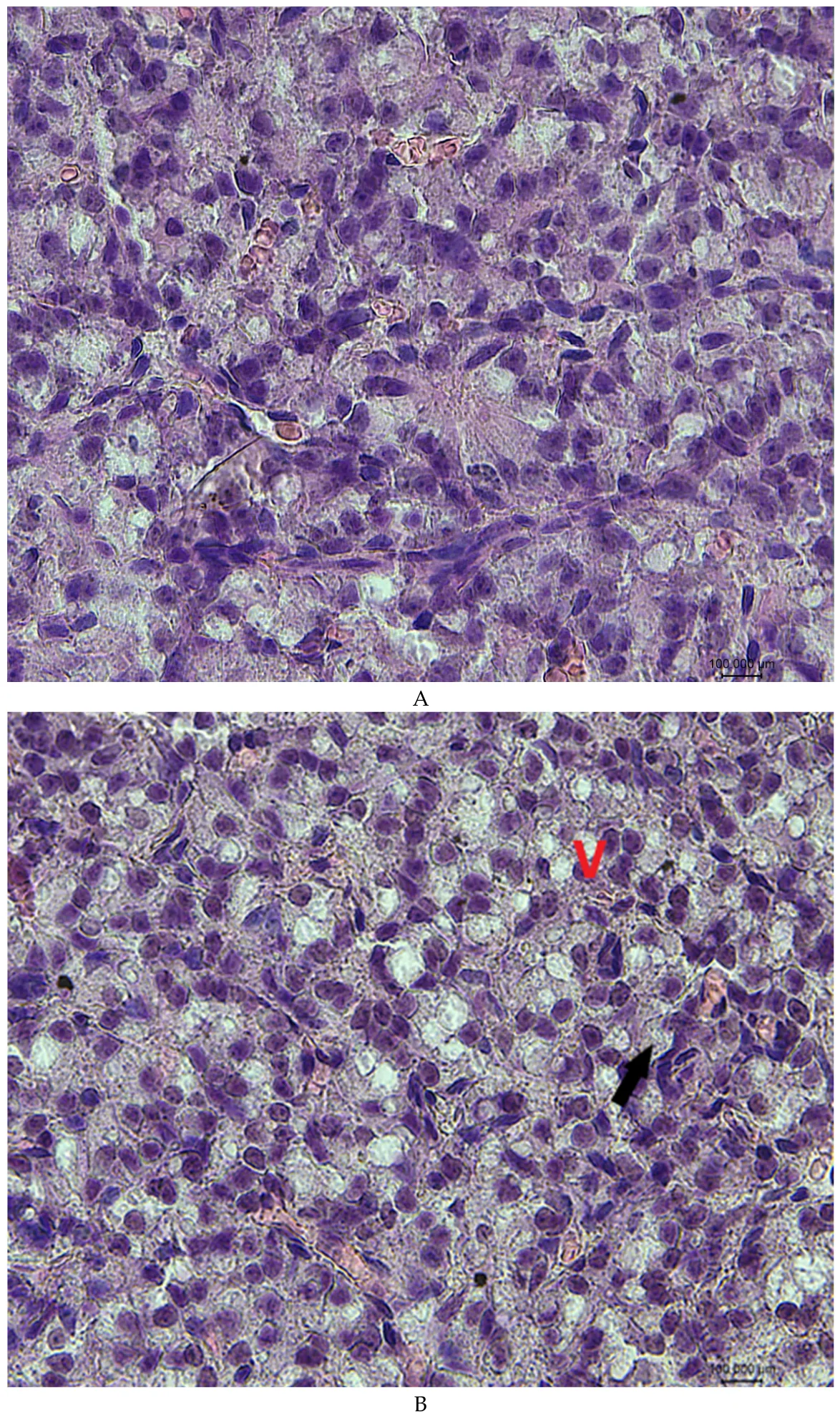

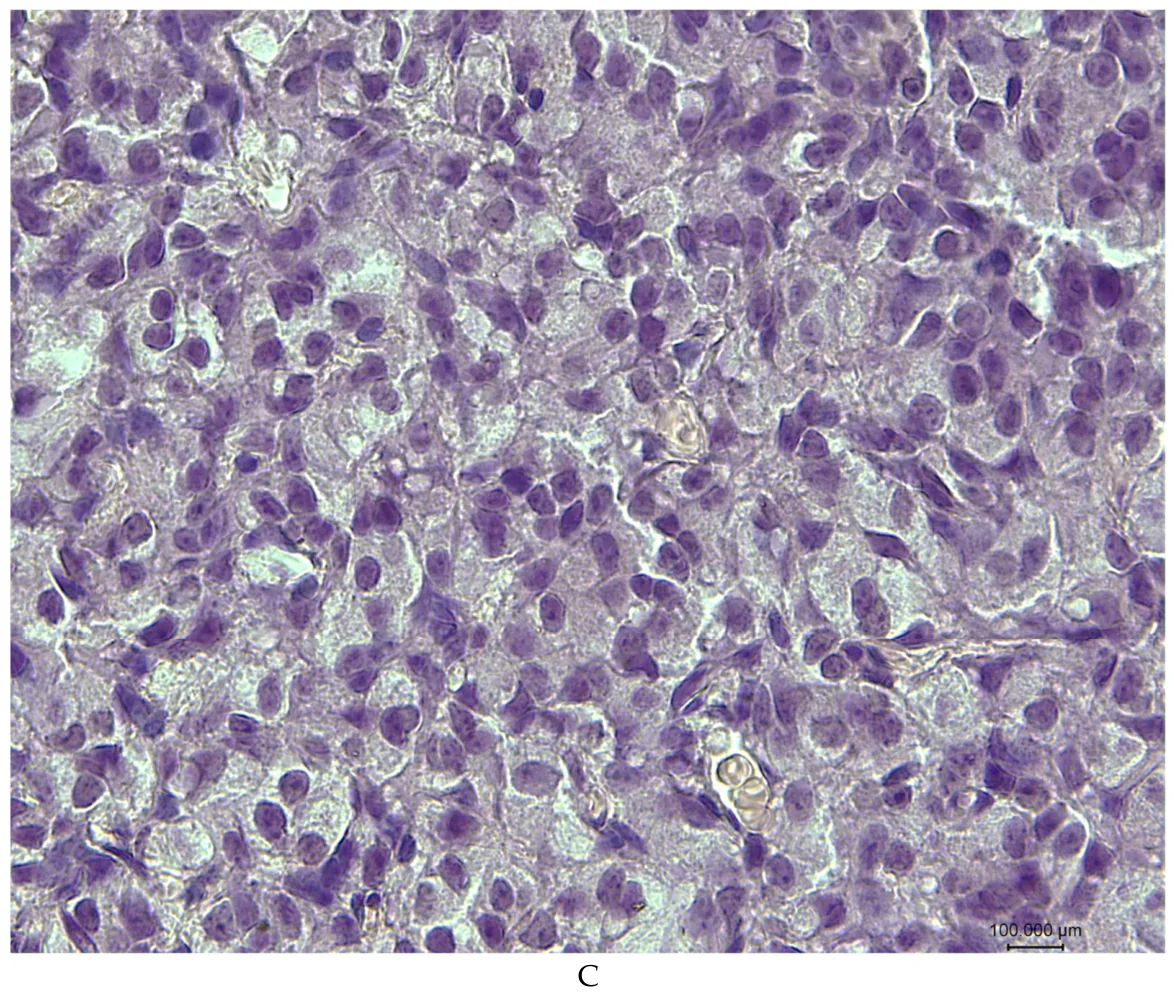
Structure of the pineal gland under light microscopy: (**A**) LD; (**B**) LL (constant light); (**C**) ICL (intermittent constant light/recovery regimen). V—vacuoles in pinealocytes; arrow—cells with morphological features characteristic of necrosis. Hematoxylin and eosin staining, ×400.

Under light microscopy, the structure of the pineal gland in animals of the control group corresponded to the norm: the organ had a pear-shaped or ovoid shape, was covered by a connective tissue capsule, from which trabeculae extended inward, forming a stroma with an abundant sinusoidal-type capillary network. The parenchyma consisted predominantly of polygonal or process-bearing pinealocytes with large, pale, round nuclei and basophilic cytoplasm (Figure 2A).

In animals of Group LL (constant lighting), vacuoles in pinealocytes were noted, and occasional cells with morphological features characteristic of necrosis were encountered (Figure 2B). The number of pinealocyte profiles per 1 mm^2^ decreased from 785.2 ± 54.7 cells/mm^2^ in the control to 646.6 ± 62.1 cells/mm^2^. In Group ICL (intermittent regimen), this value was 800.4 ± 72.84 cells/mm^2^, which did not differ from the control but was significantly higher than in the LL group (Figure 2C).

The ratio of light-to-dark pinealocytes in the control was 70.8%:29.2%. In both experimental groups, a statistically significant increase in the proportion of dark cells was recorded: to 34.6% under constant lighting and to 37.1% under the intermittent regimen (Table 1).

### 2.3. Ultrastructural Organization of Pinealocytes

#### 2.3.1. Control Group

**Figure 3:**
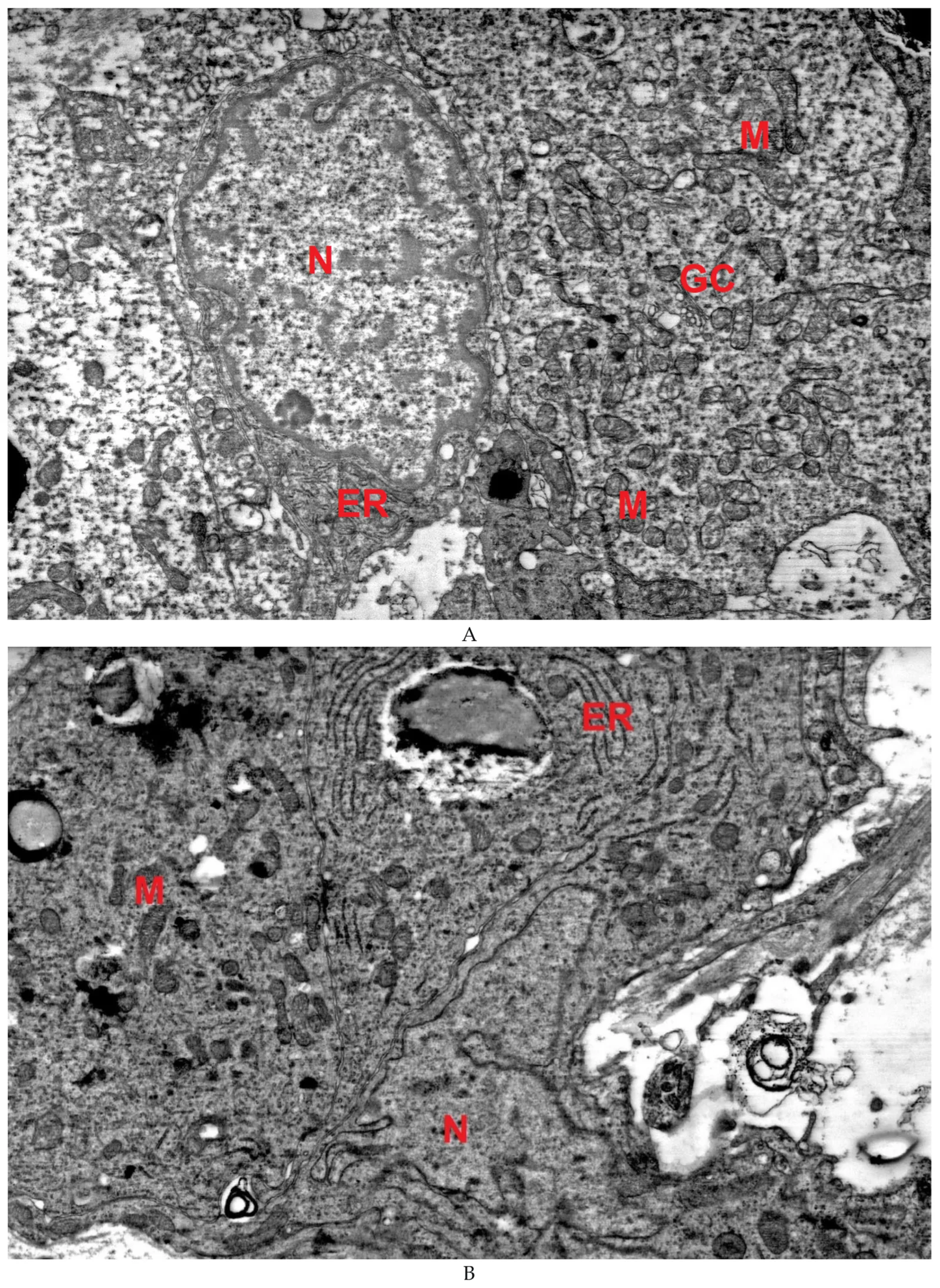
Ultrastructure of the pineal gland in control group. N—nucleus, GC—Golgi complex, ER—endoplasmic reticulum, M—mitochondria. (**A**,**B**) ×8000. TEM.

Electron microscopy of the pineal gland in control animals revealed two types of parenchymal cells: light and dark pinealocytes. The cytoplasm of light pinealocytes contained a large number of secretory vesicles, large mitochondria with few cristae and an electron-lucent matrix, a moderately developed rough endoplasmic reticulum, and a prominent Golgi complex (Figure 3A). Dark pinealocytes were characterized by smaller linear dimensions, electron-dense cytoplasm containing numerous granules, well-developed smooth and rough endoplasmic reticulum, and many small condensed mitochondria with densely packed cristae (Figure 3B). Cells of both types contained large irregularly shaped euchromatic nuclei.

#### 2.3.2. Constant Lighting

**Figure 4:**
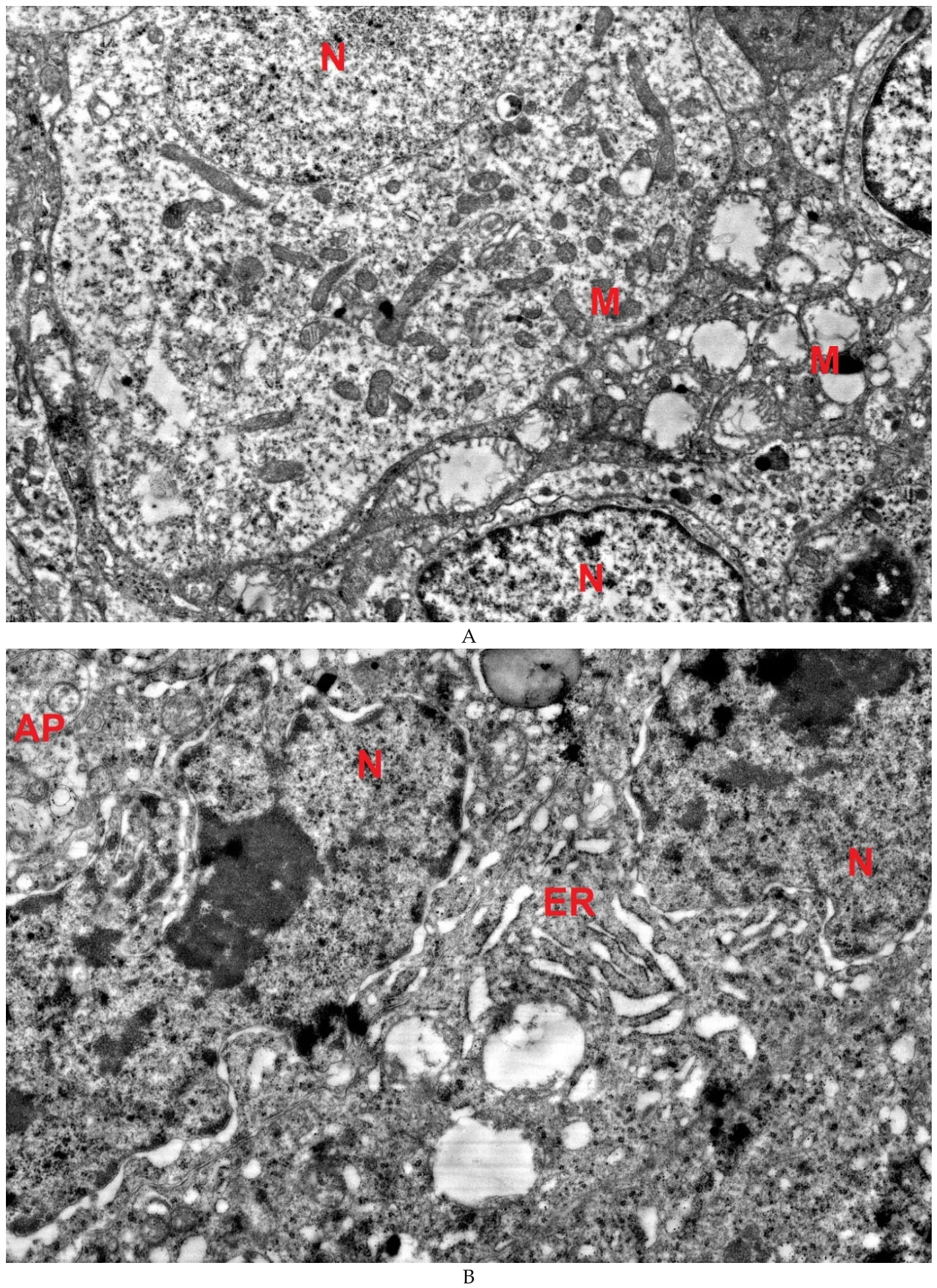

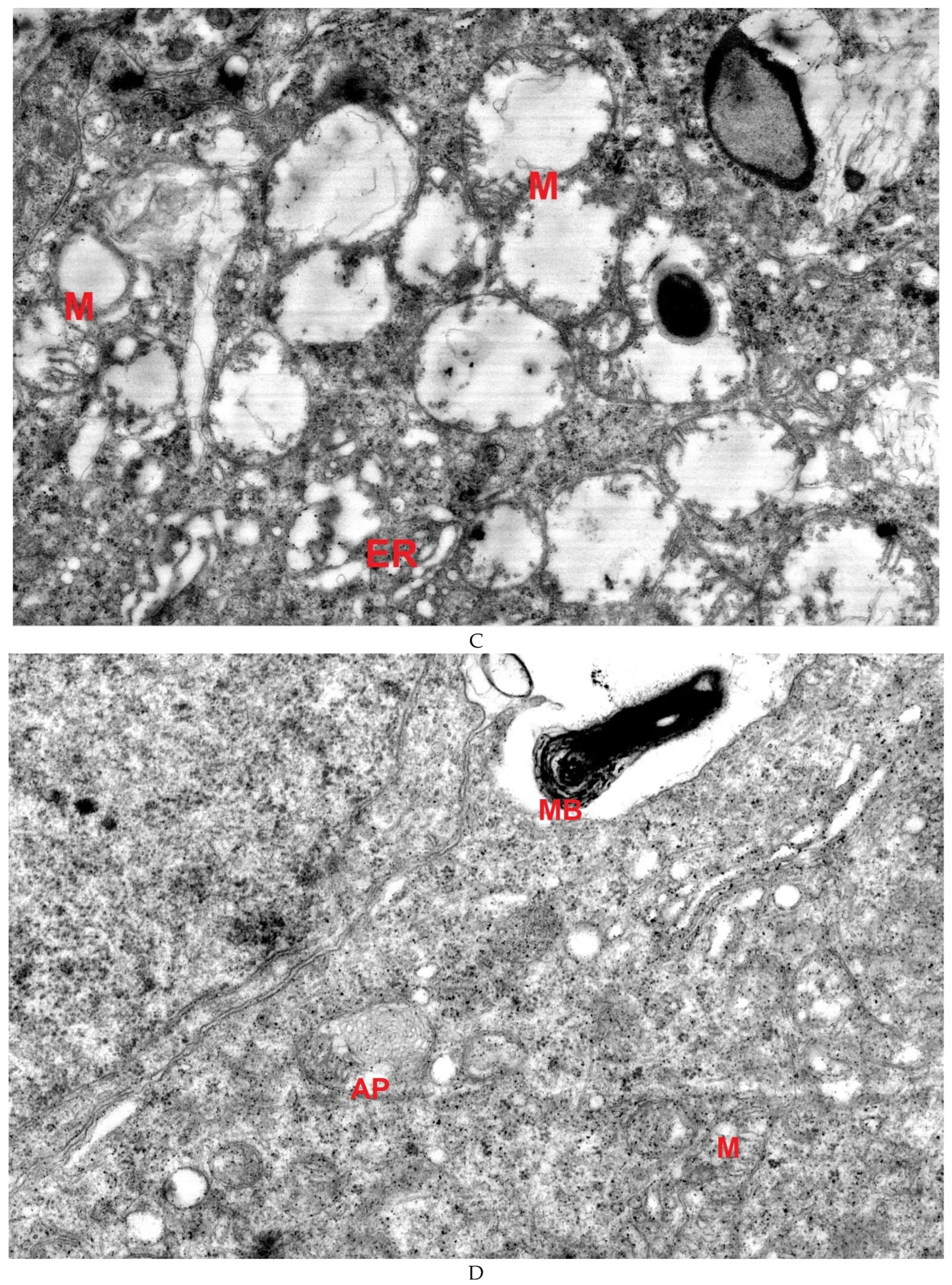
Ultrastructure of the pineal gland in Group LL. N—nucleus, AP—autophagosome, ER—endoplasmic reticulum, M—mitochondria, MB—myelin-like body. (**A**,**B**) ×8000, (**C**) ×12,000, (**D**) ×25,000. TEM.

In the pineal glands of animals in Group LL, pronounced stromal edema was noted, and the contact of dark pinealocytes with blood vessels was lost. The cytoplasm of both light and dark pinealocytes was significantly vacuolated; lipid inclusions showed signs of destruction. Similar processes affected the components of membrane organelles (endoplasmic reticulum, Golgi complex), leading to the formation of myelin-like bodies (Figure 4D). The preserved areas of membrane organelles were dilated and vacuolated (Figure 4A,C).

Pinealocyte nuclei were characterized by marked invaginations of the nuclear envelope, increased heterochromatinization, and the formation of a perinuclear rim (Figure 4B). Widespread swelling of mitochondria with vacuolization and partial destruction of cristae, and rarefaction of the matrix were observed. Newly formed mitochondria showed polymorphism of shapes with a tendency to elongation. In some cells, giant autophagosomes containing numerous organelle fragments were present (Figure 4B,C); a significant presence of individual cells with morphological features characteristic of necrosis and perivascular leukocyte infiltrates was noted.

#### 2.3.3. Intermittent Constant Light/Recovery Regimen

**Figure 5:**
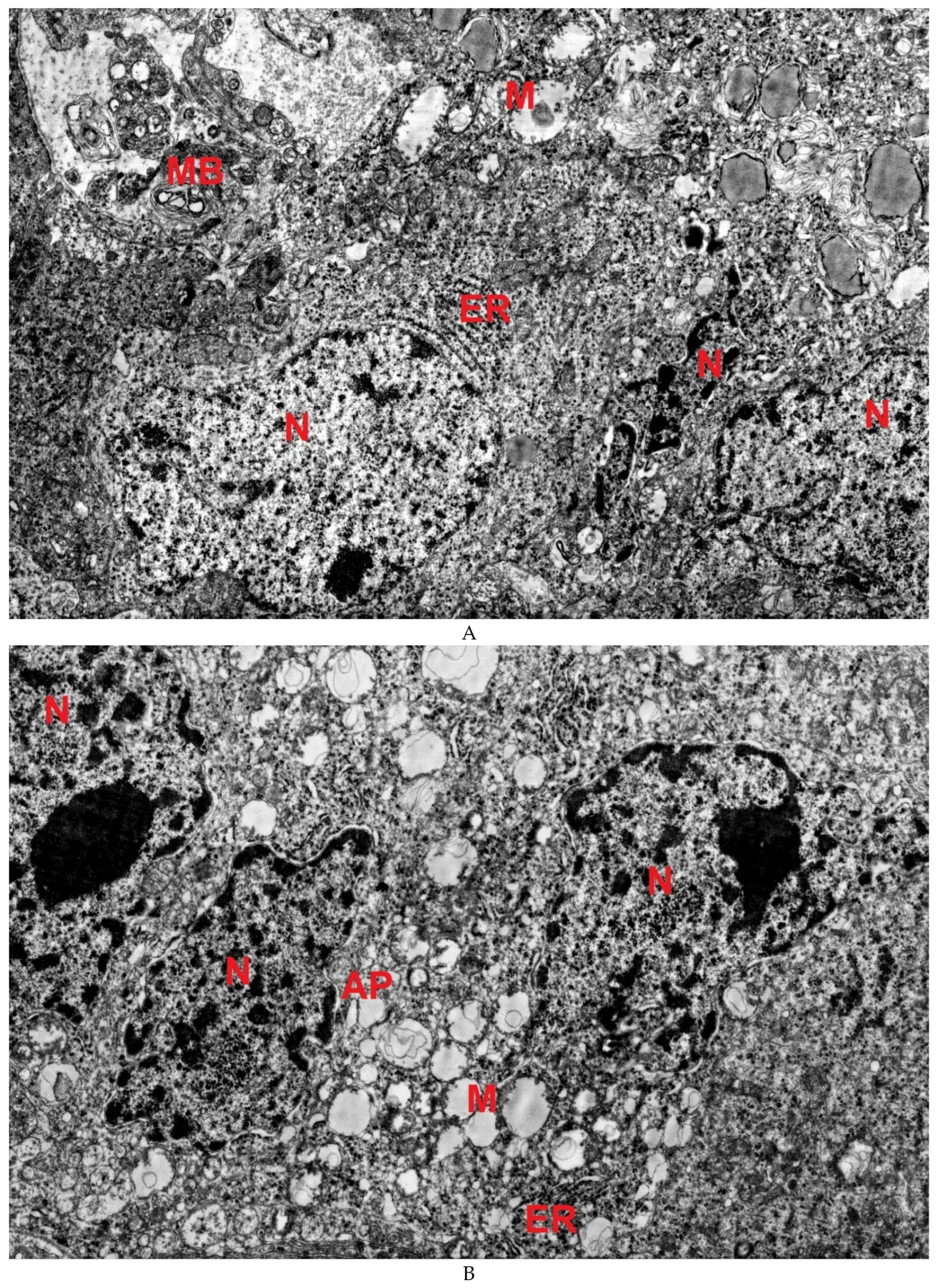

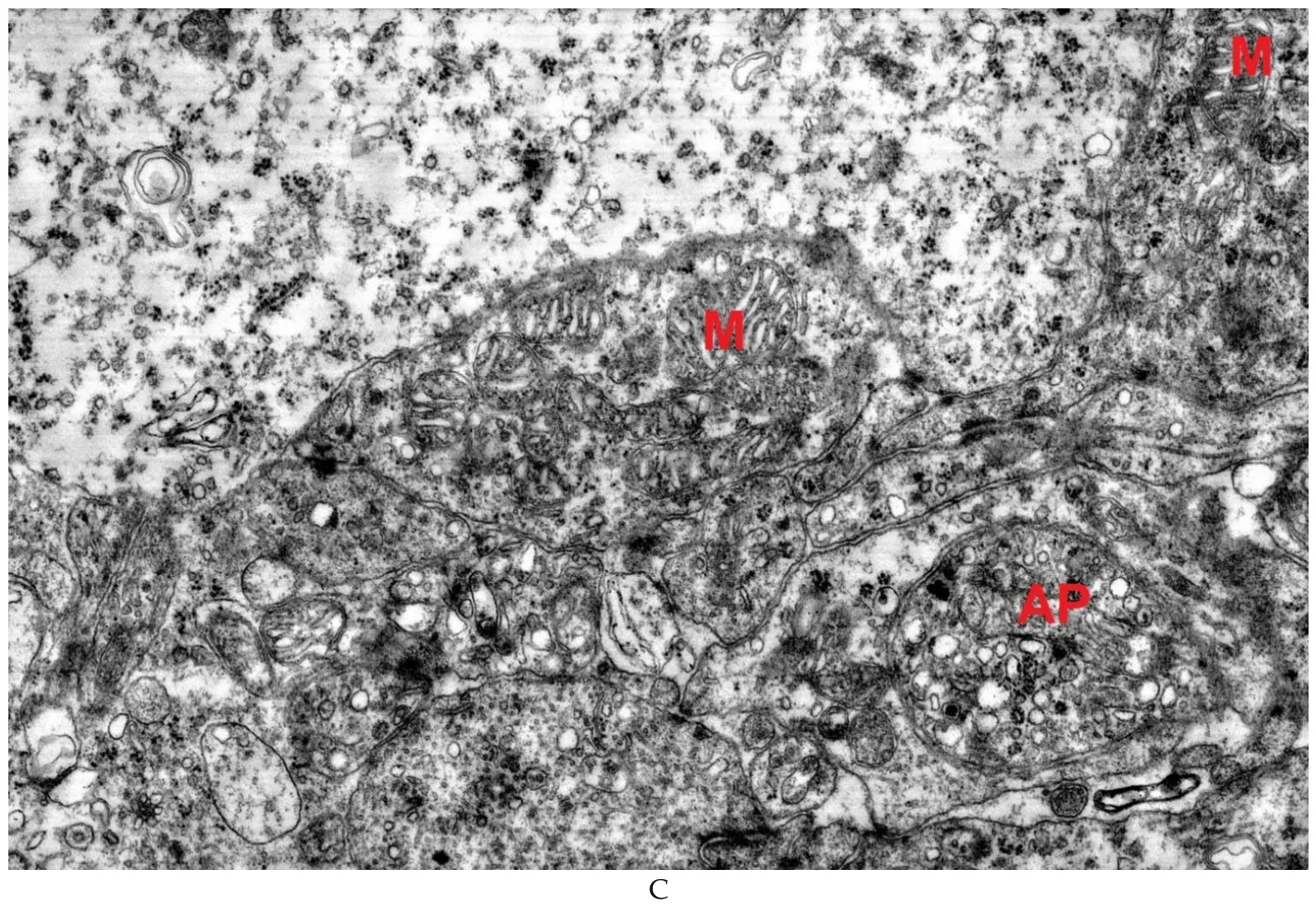
Ultrastructure of the pineal gland in group ICL. N—nucleus, AP—autophagosome, ER—endoplasmic reticulum, M—mitochondria, MB—myelin-like body. (**A**) ×6500, (**B**) ×8000, (**C**) ×18,000. TEM.

The ultrastructural picture of the pineal gland under the intermittent regimen had a number of differences. Similar but less pronounced changes in organelles were observed: fragmentation of the membranes of the endoplasmic reticulum and Golgi complex, and local dilation of cisternae (Figure 5A). However, heterochromatinization was significantly more pronounced in pinealocyte nuclei (Figure 5B). The most prominent manifestation of the intermittent regimen was changes in the ultrastructure of mitochondria: mitochondria retaining functional activity were characterized by large size, extremely low electron density of the matrix, convoluted shape, and swelling of cristae with their local destruction and formation of membrane aggregates. The number of mitochondria with pronounced vacuolization and loss of cristae exceeded that in Group LL; however, autophagosomes were sparse (Figure 5C). Significant perivascular edema was noted, with less pronounced intercellular edema and necrotic changes.

### 2.4. Morphometric Analysis of Pinealocytes

#### 2.4.1. Light Pinealocytes

Morphometric analysis revealed significant changes in the parameters of light pinealocytes in the experimental groups (Table 2).

Constant lighting led to a decrease in nuclear area by 29.5%, nuclear perimeter by 17.3%, and cell profile area by 32.2% relative to the control. Under the intermittent regimen, nuclear area was 11% lower than the control but 26.2% higher than under constant lighting; nuclear perimeter and cell profile area did not differ from control values.

#### 2.4.2. Dark Pinealocytes

In rats of Group LL, a statistically significant decrease in nuclear area (by 16.7%) and cell profile area (by 17.3%) was observed compared with the control, while nuclear perimeter remained unchanged. In rats of Group ICL, these parameters did not differ from the control and were significantly higher than in Group LL (Table 3).

### 2.5. Stereological Analysis of Organelles in Light Pinealocytes

Stereological analysis revealed significant changes in the organelle composition of light pinealocytes in the experimental groups (Table 4).

The mitochondrial profile fraction in the cytoplasm increased 1.5-fold in both experimental groups. The Golgi complex fraction decreased by 45.9% under constant lighting and by 25.5% under the intermittent regimen. Lipid droplet area decreased by more than 50% in both groups (decreased by 51.8% under constant light and by 54.5% under the intermittent regimen). The most dramatic changes were recorded for lysosomes: their area increased by 96.7% under constant lighting and by 140% under the intermittent regimen.

### 2.6. Mitochondrial Apparatus

In the mitochondria of pinealocytes in both experimental groups, there was a decrease in profile area, a reduction in perimeter, and a decrease in the number of cristae (Table 5).

The number of cristae in mitochondria decreased by 33% under constant lighting and by 20.7% under the intermittent regimen (with this value in Group ICL being 18.3% higher than in Group LL).

### 2.7. Clock Gene Protein Abundance in Pinealocytes

Immunohistochemical analysis revealed a significant increase in the abundance of CLOCK and PER2 proteins in both experimental groups.

**Figure 6:**
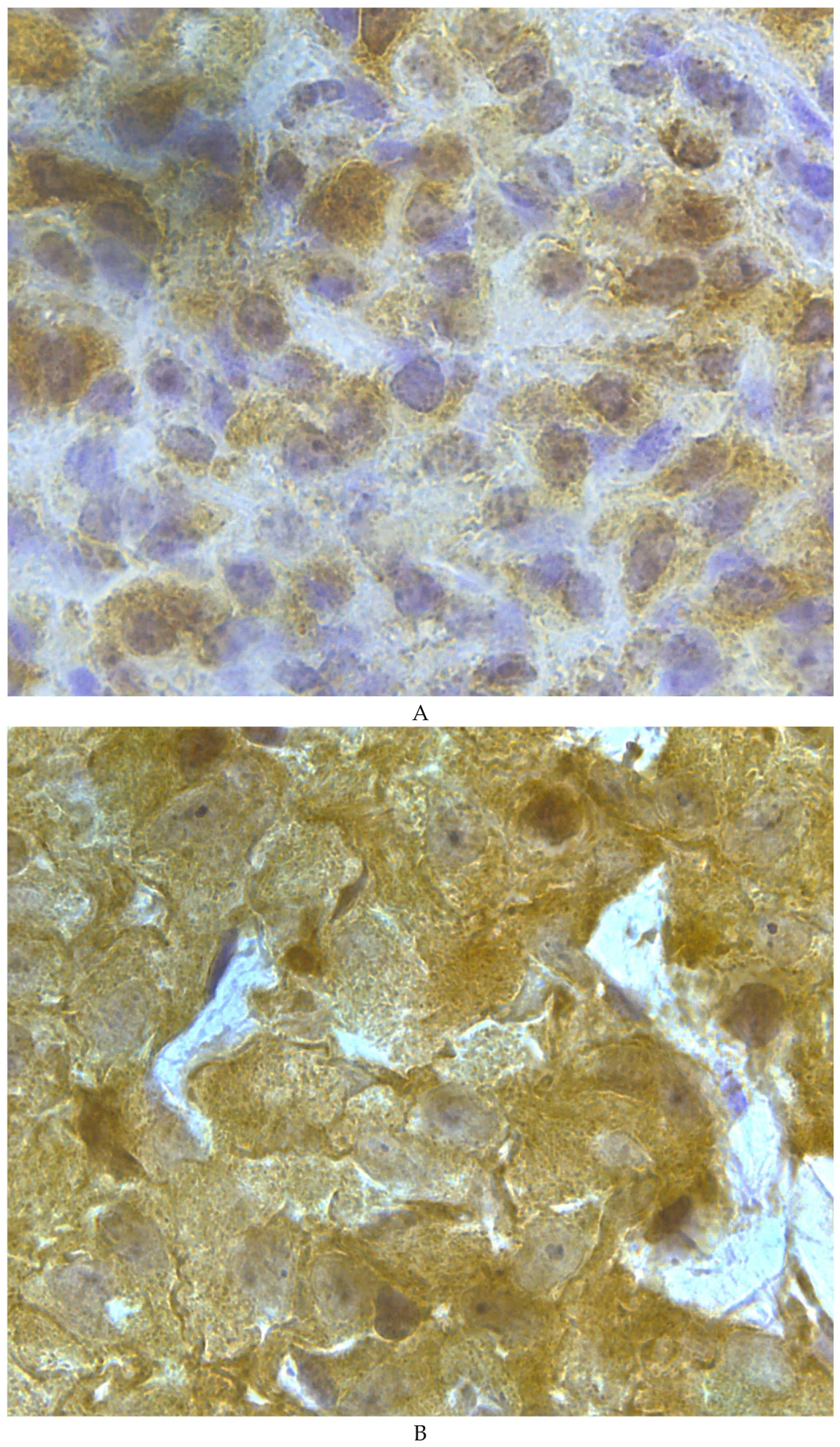

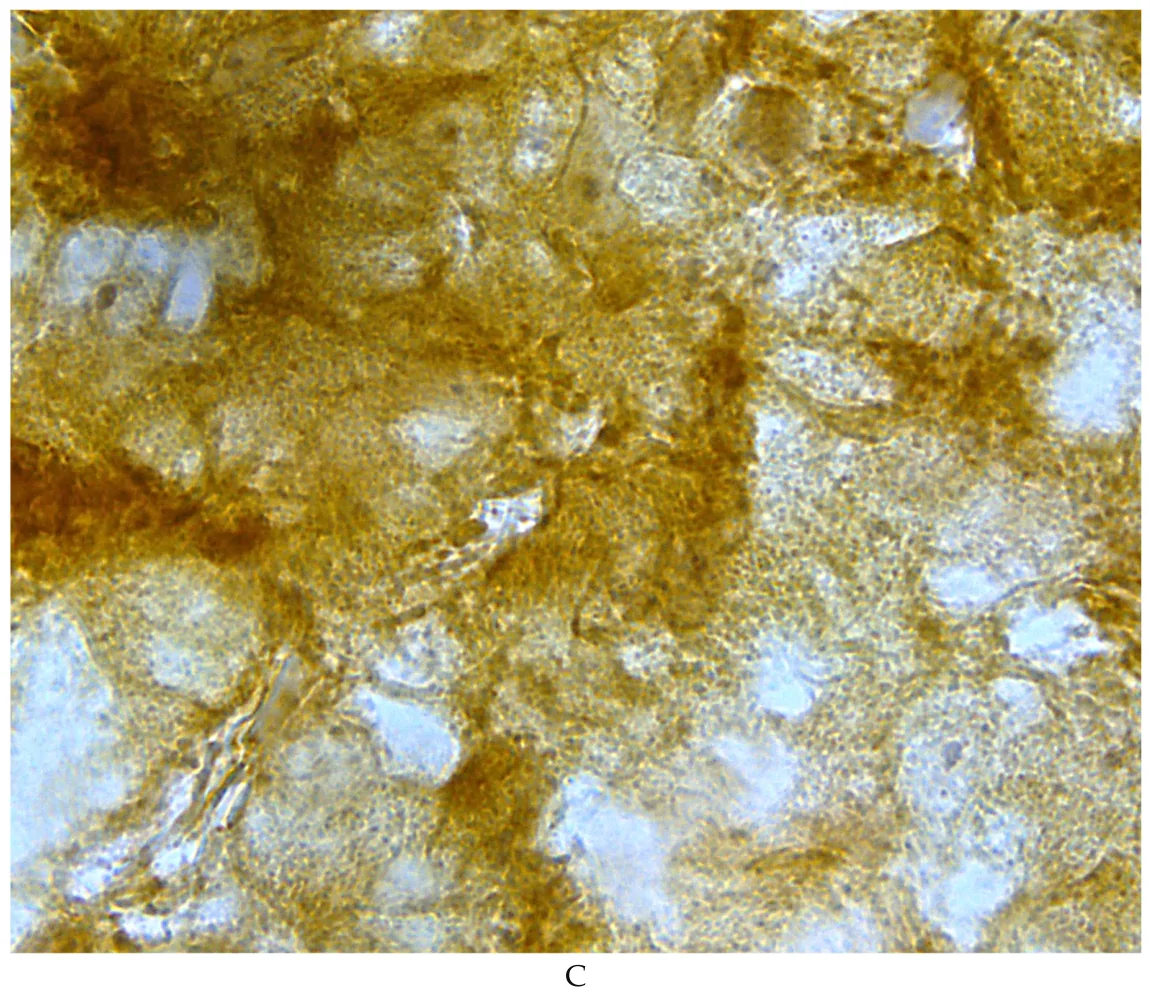
Immunohistochemical analysis of CLOCK protein immunoreactivity in pinealocytes: (**A**) LD; (**B**) LL; (**C**) ICL. ×1000.

**Figure 7:**
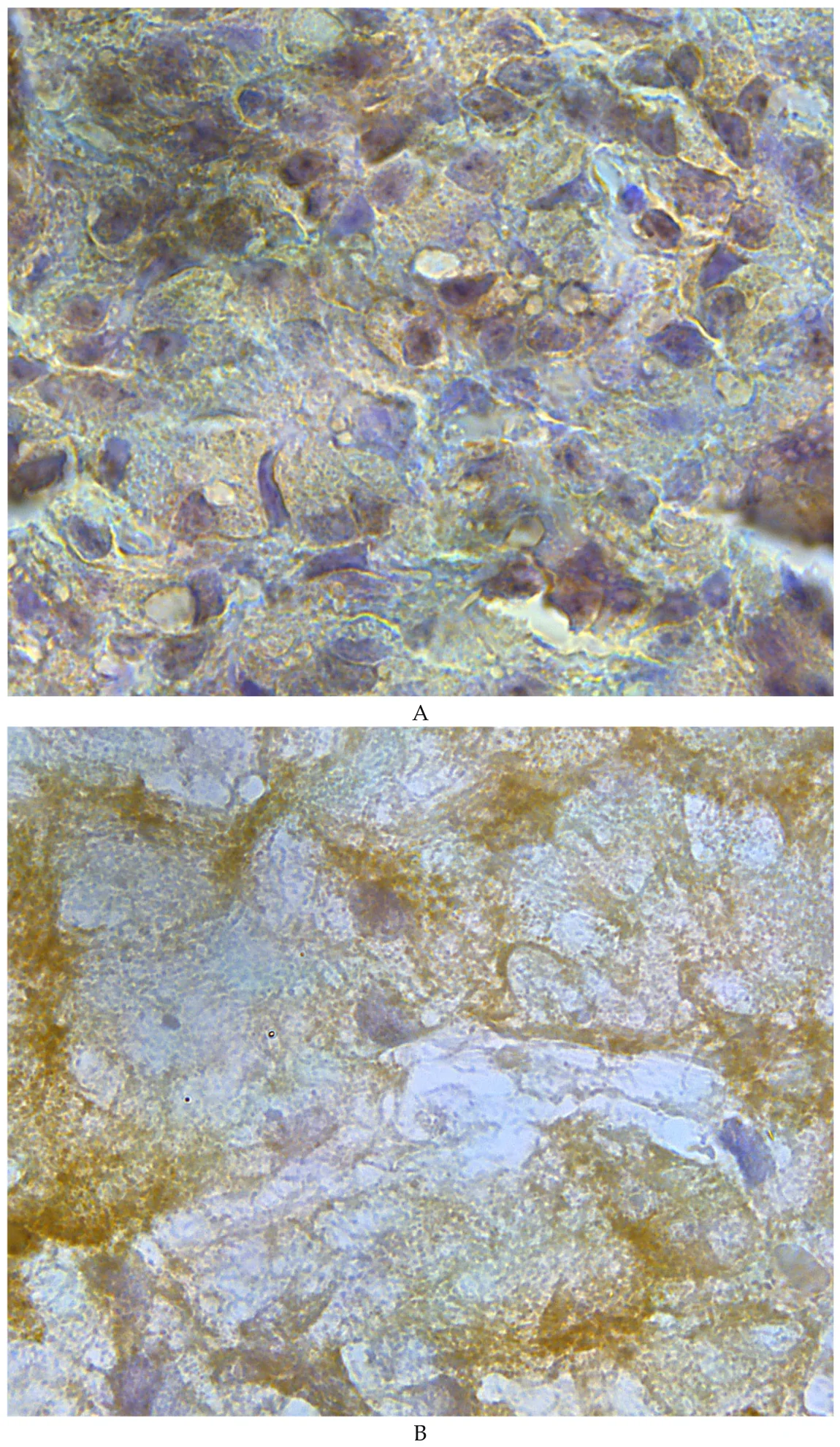

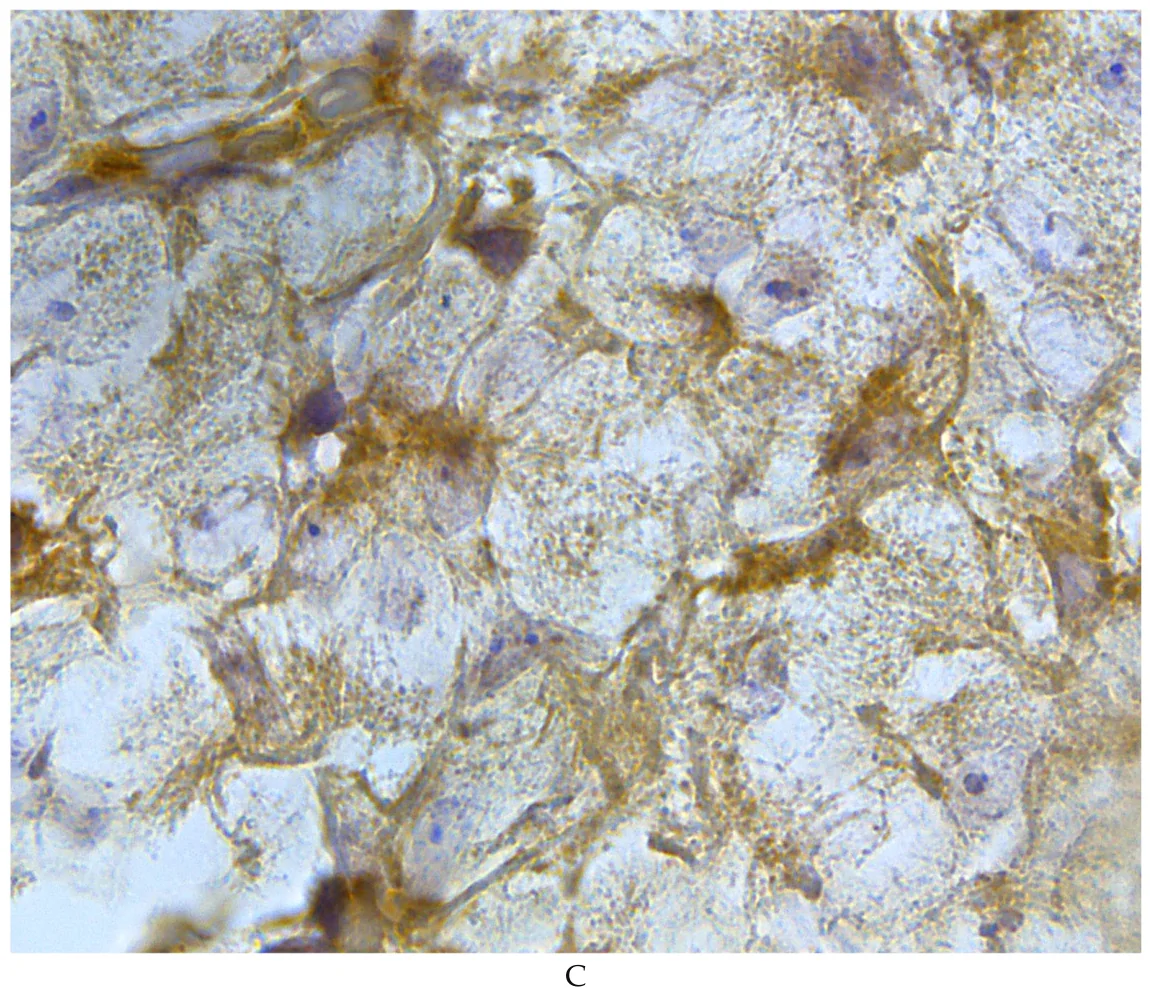
Immunohistochemical analysis of PER2 protein immunoreactivity in pinealocytes: (**A**) LD; (**B**) LL; (**C**) ICL. ×1000.

Under constant lighting, CLOCK immunoreactivity increased 1.8-fold (from 1.05 ± 0.17 to 1.88 ± 0.37 points), and PER2 immunoreactivity increased 1.8-fold (from 0.59 ± 0.18 to 1.075 ± 0.56 points). Under the intermittent regimen, PER2 immunoreactivity increased 2.9-fold (to 1.70 ± 0.49 points), and CLOCK increased 1.8-fold (to 1.91 ± 0.23 points). No significant differences in CLOCK immunoreactivity were observed between the experimental groups, whereas PER2 immunoreactivity in ICL was significantly higher than in Group LL (Figure 6 and Figure 7).

### 2.8. Blood Melatonin Levels

**Figure 8:**
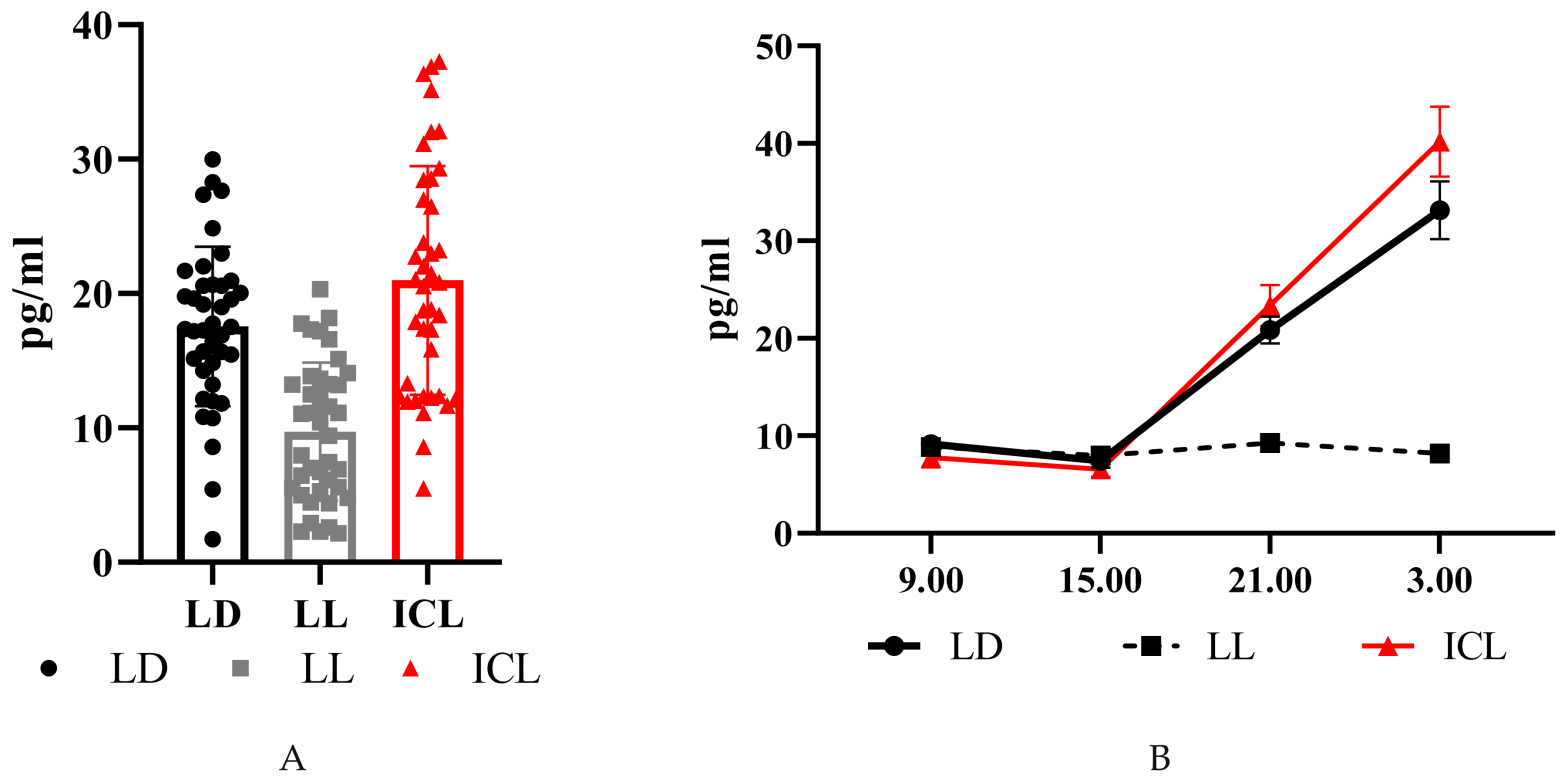
(**A**) Melatonin levels in rat blood, (**B**) daily dynamics of the melatonin levels in rat blood. Data are presented as mean ± SD; *n* = 40 per group.

The average daily blood melatonin level under constant lighting decreased from 17.65 ± 6.93 pg/mL in the control to 8.57 ± 5.96 pg/mL (Figure 8A). Under the intermittent regimen, this value was 19.46 ± 7.89 pg/mL, which did not differ from the control.

The highest concentration of melatonin, 33.15 ± 0.94 pg/mL, in Group LD was noted at 3.00, decreasing to a minimum of 7.44 ± 0.22 pg/mL by 15.00. In Group LL, the maximum value decreased to 9.25 ± 0.14 pg/mL and shifted to 21.00, with a minimum value of 7.98 ± 0.14 pg/mL, noted at 15.00. The peaks in Group ICL were noted at 3.00, with an increase in the upper limit to 40.17 ± 1.13 pg/mL and a decrease in the lower limit to 6.55 ± 0.23 pg/mL at 15.00 (Figure 8B).

### 2.9. Blood Biochemical Parameters

Both lighting regimens caused significant changes in blood biochemical parameters (Table 6).

Under constant lighting, a decrease in total protein by 18.4% and albumin by 70.1%, an increase in cholesterol by 21.9%, triglycerides by 34.1%, glucose by 29.4%, and LDH activity by 57.0% were observed. Under the intermittent regimen, changes were less pronounced for protein parameters (decrease in total protein by 9.4%, albumin by 58.1%) but more pronounced for lipid metabolism (increase in cholesterol by 33.3%, triglycerides by 48.4%) and alkaline phosphatase (increase by 58.9%). Glucose levels normalized under the intermittent regimen.

### 2.10. Body Temperature

**Figure 9:**
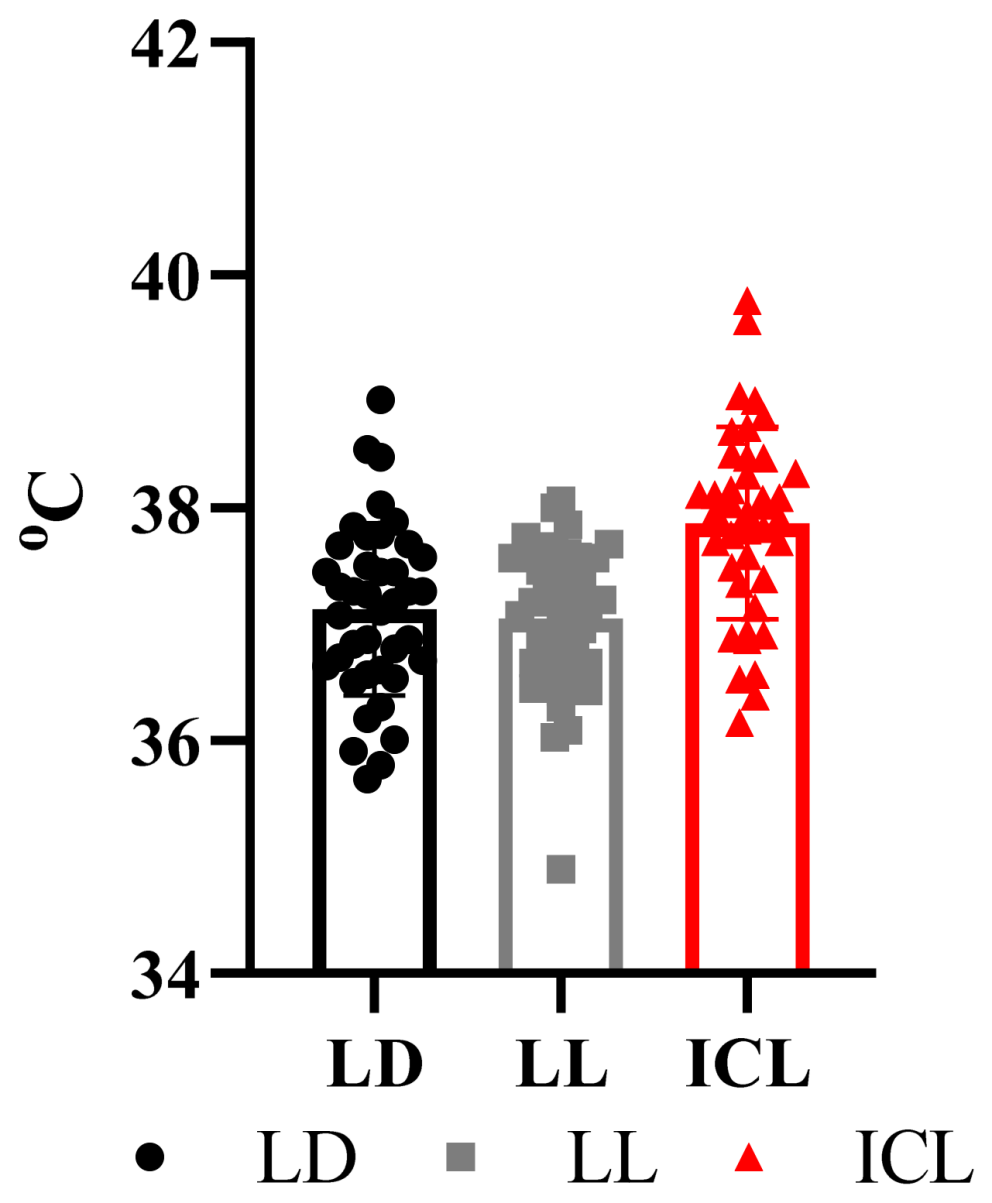
Body temperature of the rats. Data are presented as mean ± SD; *n* = 40 per group per time point.

The average daily temperature under the intermittent regimen (37.81 ± 0.72 °C) was significantly higher than in the control (37.01 ± 0.81 °C) and under constant lighting (37.15 ± 0.65 °C) (Figure 9).

### 2.11. Daily Dynamics of the Studied Parameters

#### 2.11.1. Daily Dynamic of the Body Temperature

A significant daily rhythm of body temperature was observed only in the control group, with an acrophase at 1:56 and an amplitude of 0.3 °C. In both experimental groups, no statistically detectable 24 h rhythm was present.

#### 2.11.2. Daily Dynamics of the Morphometric Parameters

In the control group, all studied morphometric parameters of light pinealocytes (nuclear area, nuclear perimeter, cell profile area) demonstrated a clear daily rhythm with a maximum in the evening hours (acrophases: 21:09, 21:50, and 21:33, respectively; amplitudes: 11,604 µm^2^, 4823 µm and 13,997 µm^2^, respectively). In Group LL, all rhythms were completely flattened. In Group ICL, rhythms were also absent; however, the parameter values were closer to the control ones.

Dark pinealocytes did not exhibit daily dynamics in any of the groups.

#### 2.11.3. Daily Dynamics of the Ultrastructural Parameters

In the control group, most ultrastructural parameters (mitochondrial fraction, number of cristae, mitochondrial area and perimeter) demonstrated significant circadian rhythms with acrophases in the evening–night hours (21:55, 22:48, 22:21, and 23:14, respectively; amplitudes: 1956 µm^2^, 1189, 0.085 µm^2^, and 0.254 µm). The Golgi complex area exhibited a rhythm with an acrophase during daytime hours (11:27) and an amplitude of 0.069 µm^2^. Lipid droplets and lysosomes did not have significant rhythms in the control.

Under constant lighting, a significant rhythm was preserved only for mitochondrial profile area (acrophase 4:26, amplitude 0.013 µm^2^), with a sharp decrease in amplitude. Under the intermittent regimen, the rhythm of mitochondrial profile area was also preserved (acrophase 17:12, amplitude 0.031 µm^2^).

#### 2.11.4. Clock Gene Protein Rhythms

In the control group, PER2 immunoreactivity had a significant rhythm with an acrophase at 15:55, and CLOCK with an acrophase at 23:44 (amplitudes: 0.08 points and 0.11 points, respectively). Under constant lighting, rhythms of both proteins were absent. Under the intermittent regimen, rhythms were preserved but with a significant shift in acrophases: for PER2—23:42, for CLOCK—11:32 (amplitudes: 0.08 points and 0.07 points, respectively).

#### 2.11.5. Melatonin Levels

In the control group, melatonin had a significant circadian rhythm with an acrophase at 1:31 and an amplitude of 14.12 pg/mL; the nighttime peak (33.15 ± 0.94 pg/mL) was 4.5 times higher than the daytime minimum. Under constant lighting, the rhythm was absent, and the maximum value decreased to 9.25 ± 0.14 pg/mL. Under the intermittent regimen, the rhythm was preserved with an acrophase at 1:28 and an amplitude of 18.54 pg/mL (30% higher than the control).

#### 2.11.6. Biochemical Parameters

The results of the cosinor analysis of biochemical parameters are presented in Table 7.

The most remarkable changes were recorded for the intermittent regimen: the amplitude of the glucose rhythm increased 5-fold (from 0.39 to 1.99 mmol/L), cholesterol—4.3-fold (from 0.21 to 0.90 mmol/L), and alkaline phosphatase—6-fold (from 61.38 to 372.65 U/L). Rhythms of LDH and creatinine disappeared in both experimental groups.

### 2.12. Multivariate Statistical Analysis

#### 2.12.1. Effect Size of the Lighting Regimen

Robust between-group analysis showed that out of the 24 studied parameters, 23 demonstrated highly significant differences between groups (*p* < 0.001). The only parameter that did not show significant differences was the nuclear perimeter of dark pinealocytes (*p* = 0.133). The effect size (η^2^) for most ultrastructural and molecular parameters exceeded 0.70, reaching 0.95 for clock gene immunoreactivity (Table 8). This means that more than 70% of the variance of these parameters is attributable to the lighting regimen.

#### 2.12.2. Pairwise Comparisons (Cohen’s d)

The most significant differences between the control and Group LL (constant light) were recorded for CLOCK immunoreactivity (g = −10.66), lipid droplet fraction (g = −8.00), nuclear area of light pinealocytes (g = −5.54), Golgi complex fraction (g = −5.56), mitochondrial fraction (g = −5.45), lysosome fraction (g = −4.95), and number of cristae (g = −4.17).

Between the control and Group ICL (intermittent regimen), the largest differences were found for PER2 immunoreactivity (g = −12.60—the largest effect in the entire study), CLOCK immunoreactivity (g = −10.15), lysosome fraction (g = −9.53), lipid droplet fraction (g = −8.50), profile area of light and dark pinealocytes (g = −3.73 and −3.76, respectively), albumin (g = −3.03), and cholesterol (g = −3.19).

Between Groups LL and ICL, the most significant differences were found for PER2 immunoreactivity (g = −5.83), Golgi complex fraction (g = −2.22), nuclear area of light pinealocytes (g = −2.14), lysosome fraction (g = −1.97), and number of cristae (g = −1.24).

#### 2.12.3. Multivariate Analysis

PCA showed that the first two principal components explained 63.7% of the total variance (PC1—48.3%, PC2—15.4%). A clear separation of the three groups along PC1 was observed, indicating qualitatively different integral phenotypes. The greatest contributions to PC1 were made by ultrastructural parameters (nuclear area of light pinealocytes, number of cristae, Golgi complex fraction), clock gene immunoreactivity, and lipid metabolism parameters.

PERMANOVA confirmed statistically significant differences between the integral phenotypes of the groups (F = 68.42, *p* = 0.001, R^2^ = 0.539), i.e., 53.9% of the total variance in the multivariate space is explained by group affiliation. Pairwise contrasts were also significant: between control and Group LL (R^2^ = 0.590), control and Group ICL (R^2^ = 0.488), and Groups LL and ICL (R^2^ = 0.271).

A regularized multiclass logistic regression model (elastic net) achieved 100% classification accuracy in all three groups (accuracy = 1.000, balanced accuracy = 1.000, multiclass AUC = 1.000), confirming the possibility of complete differentiation of the three lighting regimens based on the set of studied parameters.

#### 2.12.4. Correlation Analysis

Correlation analysis revealed a transformation of intra-system relationships depending on the lighting regimen. In the control group, physiological correlations between structural and functional parameters predominated: nuclear area positively correlated with cell profile area (rho = 0.78) and nuclear perimeter (rho = 0.72); the number of cristae positively correlated with the Golgi complex fraction (rho = 0.65) and negatively with the lysosome fraction (rho = −0.58).

Under constant lighting, the correlation structure disintegrated: the strongest negative correlation was found between the number of cristae and the lysosome fraction (rho = −0.83); PER2 immunoreactivity positively correlated with the lysosome fraction (rho = 0.76) and negatively with the nuclear area of light pinealocytes (rho = −0.71).

Under the intermittent regimen, a partial restoration of correlations was observed, but new pathological connections emerged: PER2 immunoreactivity positively correlated with the lysosome fraction (rho = 0.89—the strongest correlation), CLOCK immunoreactivity correlated with cholesterol (rho = 0.73) and triglycerides (rho = 0.69); and melatonin levels negatively correlated with PER2 immunoreactivity (rho = −0.67).

## 3. Discussion

The present comprehensive study represents the first attempt at simultaneous assessment of morphological, ultrastructural, molecular-genetic, and biochemical parameters of the rat pineal gland under two qualitatively different types of light regimen disruption. The obtained results convincingly demonstrate that constant lighting and the intermittent constant light/recovery regimen cause pronounced damaging effects on the pineal gland; however, the nature and depth of these changes are fundamentally different, allowing the distinction of two types of light stress: direct damaging (degenerative) and desynchronizing.

It is important to clarify the experimental model used in this study. The intermittent constant light/recovery regimen (24 h of constant light followed by 48 h of normal photoperiod 10:14 h light:dark, repeated cyclically) was designed to model fragmented exposure to nighttime illumination with intermittent recovery periods. This pattern is characteristic of some forms of irregular shift schedules or intermittent night work, where periods of light exposure at night are interspersed with days of normal schedule. However, it should be emphasized that this model does not constitute a full simulation of classical shift work, which typically involves systematic phase shifts, altered sleep–wake schedules, and changes in activity/feeding patterns. Rather, our model captures the effects of repeated acute light deprivation episodes with intervening recovery phases, allowing investigation of the cumulative impact of intermittent photic stress on pineal structure and function.

### 3.1. Constant Lighting as a Direct Damaging Factor

Constant lighting led to a sharp suppression of melatonin secretion, the key pineal hormone. This suppression is a direct consequence of the deprivation of the dark phase of the day, during which melatonin synthesis normally occurs. The key molecular link is arylalkylamine N-acetyltransferase (AANAT), the rate-limiting enzyme of melatonin synthesis. Classical studies have established that AANAT activity in the rat pineal gland is normally tens of times higher at night than during the day, whereas constant lighting completely suppresses the nocturnal rise in enzyme activity [35,36,37]. The light signal perceived by the retina and transmitted via the retinohypothalamic tract to the suprachiasmatic nuclei of the hypothalamus suppresses noradrenergic stimulation of pinealocytes, leading to a decrease in intracellular cAMP concentration, inhibition of protein kinase A activity, and repression of *Aanat* gene transcription [38]. Constant lighting prolongs this light-induced blockade throughout the entire daily cycle.

Our data on the complete disappearance of the melatonin rhythm and a more than 3.5-fold reduction in its maximum level are consistent with studies showing that prolonged constant lighting disrupts the daily pattern of transcription of genes encoding key melatonin synthesis enzymes and is accompanied by a significant decrease in serum melatonin levels [39,40].

The morphological equivalents of this suppression were profound degenerative changes in pinealocytes. A 30% decrease in nuclear area, 17% in nuclear perimeter, and 32% in cell profile area reflect the suppression of transcriptional and synthetic activity. Ultrastructurally, this was accompanied by pronounced heterochromatinization, formation of a perinuclear ring of condensed chromatin, and invaginations of the nuclear envelope—signs typically associated with reduced cellular synthetic activity [41].

Particular attention should be paid to the changes in the mitochondrial apparatus. The mitochondrial fraction in the cytoplasm increased 1.5-fold; however, this increase occurred against a background of a 38.9% decrease in the average area of a single mitochondrion and a 33% reduction in the number of cristae. This pattern—an increased mitochondrial fraction coupled with decreased size and structural disorganization—is characteristic of mitochondrial dysfunction that may develop under conditions of oxidative stress. Melatonin, whose synthesis is sharply suppressed under constant lighting, is a powerful mitochondria-targeted antioxidant: it not only directly scavenges reactive oxygen species within mitochondria but also stimulates the activity of electron transport chain complexes [42,43]. We hypothesize that melatonin deficiency may deprive mitochondria of this protection, potentially leading to oxidative damage to cristae membranes and impairment of oxidative phosphorylation [44]. However, direct measurements of reactive oxygen species (ROS) and oxidative stress markers (e.g., malondialdehyde, 8-hydroxydeoxyguanosine, protein carbonyls) are required to confirm this hypothesis.

The 45.9% suppression of the Golgi complex is among the most pronounced changes among all ultrastructural parameters. This reflects a profound suppression of the secretory activity of pinealocytes and is fully consistent with the almost complete absence of melatonin secretion. The suppression of the Golgi complex may be both a direct consequence of reduced precursor protein synthesis and a result of impaired vesicular transport due to ATP deficiency potentially caused by mitochondrial dysfunction [45]. However, direct measurements of ATP levels and secretory activity would be needed to confirm this mechanism.

The 96.7% increase in the lysosomal fraction and the presence of numerous autophagosomes indicate intensification of autophagy aimed at removing damaged mitochondria and other cellular structures. We speculate that the accumulation of autophagosomes with a relatively moderate increase in lysosomes could point to impaired autophagosome-lysosome fusion or reduced lysosomal hydrolase activity, potentially resulting in incomplete autophagy [46]. However, this interpretation remains hypothetical; dynamic flux studies (e.g., using lysosomal inhibitors such as chloroquine or bafilomycin A1) would be required to assess autophagic flux and distinguish between enhanced autophagosome formation and impaired degradation.

Constant lighting caused a 1.8-fold increase in the immunoreactivity of clock proteins CLOCK and PER2 with a complete loss of daily rhythmicity. Such a pattern—increased basal protein abundance with loss of rhythmicity—is characteristic of desynchronosis at the molecular level [47,48]. The mechanism of this phenomenon may be related to the uncoupling of the central pacemaker (suprachiasmatic nuclei) and the peripheral oscillator of the pineal gland. Under constant lighting, the circadian rhythmicity of the suprachiasmatic nuclei is suppressed, the nocturnal peak of norepinephrine is absent, and pinealocytes are deprived of the external synchronizing signal. We suggest that the increased abundance of CLOCK and PER2 could reflect a non-specific cellular stress response to oxidative damage and melatonin deficiency, since both proteins are involved in the regulation of the cell cycle, apoptosis, and the response to oxidative stress [49,50]. However, functional studies (e.g., transcriptional activity assays or chromatin immunoprecipitation) would be required to determine whether increased protein abundance translates into enhanced functional activity of the clock complex.

### 3.2. Intermittent Constant Light/Recovery Regimen as a Desynchronizing Factor

The most intriguing result is the absence of a significant decrease in the average daily melatonin level under the intermittent regimen, with a tendency towards some increase and a 30% increase in rhythm amplitude. This paradoxical phenomenon may be explained by a rebound effect—compensatory hyperactivation of melatonin synthesis following acute light suppression. Studies on avian pineal cell cultures have shown that after daytime light exposure, a higher level of nocturnal melatonin production is observed, interpreted as a rebound effect after cessation of light exposure [51,52]. In our experiment, animals of Group ICL, after 24 h of constant lighting, were returned to a normal photoperiod for 48 h. This time may have been sufficient for the activation of noradrenergic stimulation of pinealocytes during the dark phases and compensatory accumulation of melatonin precursors during periods of darkness after short-term deprivation. The contribution of extrapineal melatonin synthesis, which could partially compensate for pineal dysfunction, cannot be excluded [53]. However, direct measurements of AANAT activity and melatonin precursors (e.g., N-acetylserotonin) would be required to confirm this rebound mechanism.

As noted above, this model reflects fragmented exposure to nighttime illumination with recovery periods, characteristic of some forms of irregular shift patterns, rather than a complete model of shift work with systematic phase shifts. The intermittent nature of the light exposure in our paradigm allows investigation of the cumulative effects of repeated photic stress episodes and the adequacy of recovery periods between them.

However, despite the preservation and even enhancement of the melatonin rhythm, the intermittent regimen caused the most pronounced changes in a number of parameters. The lysosomal fraction increased 2.4-fold—this was the highest increase among all groups, indicating hyperactivation of the lysosomal system. At the same time, the number of autophagosomes was lower than in Group LL, which may suggest preservation of autophagic flux and active degradation of damaged components. We hypothesize that excessive lysosomal activation under the intermittent regimen could contribute to the triggering of lysosome-mediated apoptosis [54]. However, this remains a hypothesis requiring experimental verification; direct measurements of lysosomal enzyme activity (e.g., cathepsin B, cathepsin D), autophagic flux, and apoptotic markers (e.g., caspase-3 activation, TUNEL assay, Annexin V staining) are needed to confirm these interpretations.

The number of cristae in mitochondria under the intermittent regimen, although 20.7% lower than the control, was 18.3% higher than under constant lighting. This indicates partial recovery of mitochondrial function. However, ultrastructurally, mitochondria in Group ICL showed signs of both damage and activation of compensatory processes: large mitochondria with low electron density of the matrix, convoluted shape, and swollen cristae. The number of mitochondria with pronounced vacuolization and loss of cristae in this group exceeded that under constant lighting, suggesting a more intense process of mitochondrial damage, possibly due to repeated cycles of suppression and restoration of melatonin synthesis. Such a phenomenon is known for other tissues, where intermittent exposure to a stressor can cause more severe damage than continuous exposure [55]. It should be emphasized that these interpretations are speculative and require direct experimental confirmation through measurements of mitochondrial function (e.g., oxygen consumption rate, membrane potential, ATP production) and oxidative damage markers.

Clock protein abundance under the intermittent regimen deserves special attention. PER2 immunoreactivity increased 2.9-fold—the highest increase among all groups, while rhythmicity was preserved but with a shift in acrophase. Why was PER2 abundance under the intermittent regimen higher than under constant lighting? We hypothesize that this is related to the need for constant re-adaptation of the molecular clock to the changing regimen. The PER2 protein is a key component of the negative feedback loop, and its pronounced induction is necessary for efficient suppression of CLOCK/BMAL1-dependent transcription and closure of the circadian cycle [22]. With alternating phase shifts caused by intermittent constant lighting, the circadian clock system may undergo repeated stress, requiring enhanced PER2 expression to “reset” and re-adjust the phase. This resembles the situation with jet lag, where PER expression in the suprachiasmatic nuclei and peripheral tissues sharply increases during resynchronization [56].

In addition, the PER2 protein performs not only chronometric functions but also participates in the regulation of metabolism by interacting with nuclear receptors PPARγ and Rev-erbα [57,58]. The strong positive correlation between PER2 immunoreactivity and the lysosomal fraction found in Group ICL (rho = 0.89) warrants separate discussion. We propose that PER2 may regulate autophagy through interaction with transcription factors controlling the expression of autophagy genes, or through modulation of mTOR signaling pathway activity [59,60]. Hyperactivation of this link under intermittent regimen conditions could reflect the formation of a potential vicious cycle: desynchronization → PER2 upregulation → enhanced autophagy → altered amino acid pool → further disruption of clock regulation. Again, this mechanistic model remains hypothetical and requires functional studies, such as PER2 knockdown or overexpression experiments, to establish causality.

### 3.3. Metabolic Consequences

The identified changes in the pineal gland were predictably reflected in systemic metabolic parameters. Under constant lighting, hyperglycemia (increase by 29.4%), dyslipidemia (increase in cholesterol by 21.9%, triglycerides by 34.1%), hypoproteinemia, and hypoalbuminemia were observed. These changes are consistent with data on the development of insulin resistance and impaired glucose tolerance under conditions of dark deprivation [17,61].

Under the intermittent regimen, despite normalization of the average daily glucose level, the amplitude of its rhythm increased 5-fold. This indicates that the intermittent regimen does not cause persistent hyperglycemia but destabilizes glycemic control, making it excessively dependent on the time of day. Such “glycemic lability” could be a predictor of subsequent diabetes development with continued exposure.

Lipid metabolism disturbances under the intermittent regimen were even more pronounced than under constant lighting: cholesterol increased by 33.3%, triglycerides by 48.4%, and the amplitudes of their rhythms increased 4.3-fold. This may be related to hyperactivation of PER2, which, as mentioned, modulates lipid metabolism. It has been shown that overexpression of *Per2* in the mouse liver leads to the development of steatosis and hypertriglyceridemia [62].

The most striking distinguishing feature of the intermittent regimen was the behavior of alkaline phosphatase. While its activity did not change under constant lighting, under the intermittent regimen, it increased by 58.9%, and the amplitude of its rhythm increased 6-fold. Isolated elevation of alkaline phosphatase under the intermittent regimen could indicate specific damage to the hepatobiliary system, characteristic of intermittent stress. Existing literature describes an increase in alkaline phosphatase in experimental desynchronosis in rodents, linked to cholestasis potentially caused by disrupted rhythmic expression of bile acid transporters in hepatocytes. However, confirmation of this mechanism would require direct measurements of bile acid levels and hepatic transporter expression.

### 3.4. Two Types of Light Stress: Comparative Analysis

Based on our analysis, we propose to distinguish two types of effects of disrupted light regimens on the pineal gland:

1. Direct damaging (degenerative) effect (constant lighting)
  ○ Profound atrophy of light pinealocytes (decrease in nuclear area by 30%, cell area by 32%);
  ○ Suppression of the secretory apparatus (reduction of the Golgi complex by 1.8-fold);
  ○ Mitochondrial dysfunction with loss of cristae (decrease in cristae number by 33%) and compensatory swelling;
  ○ Complete suppression of melatonin secretion with disappearance of its rhythm;
  ○ Constant, arrhythmic overexpression of clock proteins;
  ○ Loss of circadian rhythms for most biochemical parameters and body temperature.

Leading markers of this type of damage: Golgi complex state, nuclear area of light pinealocytes, and number of cristae in mitochondria.

2. Desynchronizing effect (intermittent constant light/recovery regimen)
  ○ Partial preservation of pinealocyte structure (nuclear area reduced by only 11%, cell area close to normal);
  ○ Preservation of melatonin secretion with a paradoxical increase in rhythm amplitude;
  ○ Pronounced activation of the lysosomal system (2.4-fold increase in lysosomal fraction);
  ○ Hyperactivation of clock proteins, especially PER2 (2.9-fold increase), with preservation of rhythmicity but shifted acrophases;
  ○ Pronounced metabolic disturbances (4.3-fold increase in amplitude of cholesterol rhythm, and 6-fold for triglycerides and alkaline phosphatase) with normal glucose levels;
  ○ Loss of body temperature and LDH rhythms.

Leading markers of desynchronizing action: lysosomal fraction, cholesterol level, and PER2 immunoreactivity.

Multivariate statistical analysis confirmed the qualitative differences between the two types of light stress. PCA demonstrated clear separation of the three groups, and the elastic net model achieved 100% classification accuracy. This means that the set of studied parameters constitutes a reliable diagnostic tool for differentiating types of light stress.

### 3.5. Clinical Diagnostic Significance and Perspectives

The obtained results have important practical implications. The identified quantitative morphological (nuclear area of light pinealocytes, Golgi complex fraction, cristae number, lysosomal fraction) and functional (cholesterol level, PER2 and CLOCK immunoreactivity) markers can be recommended for preclinical assessment of the efficacy of adaptogens, chronobiotics (in particular, exogenous melatonin), and lighting normalization regimens.

Of particular interest is the finding that two days of normal regimen after 24 h of constant lighting are insufficient for complete recovery of the mitochondrial apparatus and normalization of nuclear architecture. This indicates the persistence of induced changes even after a short acute light deprivation and points to the need for developing longer recovery protocols.

Correlation analysis revealed a transformation of regulatory relationships under different lighting regimens: in the control, physiological correlations between structure and function predominate; under constant lighting, the correlation structure disintegrates; under the intermittent regimen, new pathological connections form between clock proteins and metabolic disturbances. This may reflect the formation of a new, pathological “homeostasis” of desynchronosis, in which the molecular clock and metabolism could become coupled in a vicious cycle. However, causal relationships between these observed correlations cannot be established from this cross-sectional study; longitudinal and interventional experiments are required.

### 3.6. Study Limitations

This study has a number of limitations that should be considered when interpreting the results.

1. Animal model and sex: The experiment was conducted exclusively on male Wistar rats. This limits the generalizability of our findings to females, as sex differences have been reported in circadian rhythms, melatonin secretion, and pineal gland function [62,63]. Estrogen and progesterone are known to modulate circadian rhythms and pineal melatonin synthesis [64]. Therefore, the observed effects may differ in female animals, and future studies should include both sexes to assess potential sex-dependent responses to light stress.
2. Experimental duration: The duration of the experiment was 21 days. While this period is sufficient to induce structural and functional changes in the pineal gland, the question of long-term consequences (over 3–6 months) and the reversibility of the observed changes remains open. It is unclear whether the degenerative changes observed under constant lighting would progress further with prolonged exposure, or whether the alterations induced by the intermittent regimen would become more pronounced or eventually adapt. Longitudinal studies with extended exposure periods and recovery phases are needed to address these questions.
3. Molecular scope: The study did not include assessment of several key molecular players in the circadian and melatonin systems, including:
  - Other clock genes: *Bmal1*, *Cry1*, *Cry2*, *Rev-erbα*, *Rorα*;
  - Melatonin synthesis enzymes: *Aanat* (arylalkylamine N-acetyltransferase) and *HIOMT* (hydroxyindole O-methyltransferase);
  - Melatonin receptors: MT1 and MT2;
  - Downstream signaling pathways: CREB, PKA, ERK.

Inclusion of these molecular markers could have provided a more complete picture of the molecular mechanisms underlying the observed structural and functional changes. Specifically, measurement of AANAT activity would have allowed direct confirmation of the hypothesized suppression of melatonin synthesis under constant lighting and the proposed rebound effect under the intermittent regimen.

4. Absence of direct functional measurements: We did not perform direct measurements of several functional parameters that are critical for interpreting our findings:

**Parameter****Why It Matters****What Would Be Needed to Confirm**Reactive oxygen species (ROS)To confirm oxidative stress as a mechanism of mitochondrial damageDirect ROS measurements (e.g., DCFH-DA, Amplex Red), lipid peroxidation markers (MDA, 4-HNE), protein oxidation markers (carbonyls, 8-OHdG)AANAT activityTo confirm suppression/rebound of melatonin synthesisEnzyme activity assay (radiometric or HPLC-based)Autophagic fluxTo distinguish between enhanced autophagosome formation and impaired degradationFlux assays with lysosomal inhibitors (chloroquine, bafilomycin A1); LC3-II turnoverApoptosisTo confirm whether cell death mechanisms are activatedCaspase-3/7 activity, TUNEL assay, Annexin V staining, cytochrome c releaseMitochondrial functionTo determine whether structural changes translate to functional impairmentOxygen consumption rate (Seahorse), mitochondrial membrane potential (JC-1), ATP levelsExtrapineal melatoninTo assess whether peripheral sources compensate for pineal dysfunctionMelatonin measurements in tissues (gut, retina, immune cells)

Therefore, all interpretations regarding these mechanisms are hypothetical in nature and require experimental verification through dedicated functional assays.

5. Lack of behavioral monitoring: The level of physical activity, sleep–wake patterns, and precise feeding/drinking patterns were not recorded. Although food and water were available ad libitum, changes in consumption patterns may be a consequence rather than a cause of desynchronization, and may contribute to the observed metabolic changes. Circadian rhythms in feeding behavior are well established [65], and disruption of these rhythms could independently affect lipid and glucose metabolism. Future studies should include continuous monitoring of locomotor activity, feeding behavior, and sleep patterns to distinguish between direct photic effects and indirect effects mediated by behavioral changes.
6. Causal relationships and mechanistic studies: Our correlation analyses revealed strong associations between various parameters (e.g., PER2 immunoreactivity and lysosomal fraction, ρ = 0.89). However, correlation does not imply causation. Experiments with genetic modification (e.g., PER2 knockout or knockdown) or pharmacological modulation (e.g., PER2 inhibitors or activators) are required to establish causal relationships between clock protein changes and the observed downstream effects, including lysosomal activation and metabolic disturbances.
7. Modeling limitations: The intermittent constant light/recovery regimen used in this study, while modeling fragmented light exposure with recovery periods characteristic of some irregular schedules, does not fully replicate the complex behavioral and physiological changes associated with shift work in humans, including:
  - Altered sleep–wake timing;
  - Changes in meal timing and composition;
  - Social and environmental stressors;
  - Extended periods of exposure (years to decades);
  - Inter-individual variability in tolerance.

Therefore, direct extrapolation of our findings to human shift work conditions should be made with caution. The model is more accurately described as a model of intermittent photic stress with recovery periods, rather than a complete model of shift work.

8. Sample size for immunohistochemistry: While the sample size for most analyses (*n* = 10 per group per time point) was sufficient for detecting the observed effects (as confirmed by power analysis), the sample size for immunohistochemical analysis was smaller (*n* = 5 per group per time point). This was necessitated by the limitations of antibody availability and staining capacity in a single batch. Although *n* = 5 is within the range commonly used in similar immunohistochemical studies [66,67], the smaller sample size may have reduced statistical power for detecting subtle differences in clock protein expression, particularly at individual time points.
9. Spectral composition of light: We did not perform independent spectroradiometric measurements of the light source. Although we report the manufacturer’s specifications (color temperature 4000 K, spectral peaks at 450–460 nm and 550–600 nm), the exact spectral power distribution and its potential variation over time were not characterized. This limits the precision with which our results can be compared to studies using different light sources, and may affect the reproducibility of our findings. Different wavelengths have different effects on melatonin suppression, and the absence of detailed spectral data limits the interpretation of our results in the context of wavelength-specific effects.
10. Statistical considerations: The cross-sectional design (different animals at each time point) did not allow assessment of intra-individual circadian variation or changes over time within the same animal. While this design was necessary for tissue collection, it limits the ability to assess the stability of rhythms at the individual level. Moreover, cosinor analysis was performed on group-averaged data, which may mask inter-individual variability in rhythm parameters.
11. Tissue processing and artifacts: As with all ultrastructural studies, the possibility of fixation and processing artifacts cannot be entirely excluded. However, all groups were processed identically, and the presence of normal ultrastructure in control samples suggests that the observed changes reflect genuine biological differences rather than technical artifacts.

## 4. Materials and Methods

### 4.1. Research Object and Conditions of Detention

The study was performed on 120 male outbred Wistar rats aged 5 months and weighing 250 ± 15 g at the start of the experiment. The animals were obtained from the nursery of the Shared Use Center “SPF-vivarium” of the Institute of Cytology and Genetics of the Siberian Branch of the Russian Academy of Sciences.

All animals were placed in standard plastic cages (5 rats per cage, dimensions 60 × 40 × 30 cm) at a temperature of 20–22 °C and a relative air humidity of 60–70%. Rats had free access to drinking water and briquetted feed PC-120-1. The cages were arranged in a single row on racks at an equal distance from the light source. The position of cages within the rack was randomized and rotated every 3 days to minimize potential positional effects [68].

All animals underwent 14 days of acclimatization under standard vivarium conditions (12:12 light:dark cycle, lights on at 8:00 AM, lights off at 8:00 PM) before the start of the experiment. During this period, animals were handled daily to minimize stress-related effects.

Lighting was provided by white LED lamps with a correlated color temperature of 4000 K and spectral peaks in the ranges 450–460 nm and 550–600 nm. Illuminance at cage level was 250 ± 15 lux, measured with a TKA-PKM luxmeter (Scientific Instruments «ТКА», St. Petersburg, Russia) at three points on each rack (top, middle, and bottom shelves) at the beginning of the experiment and every 3 days thereafter to ensure stability. During the dark phase, lighting was completely absent (0 lux). Light–dark transitions were instantaneous (automated timer). For manipulations during the dark phase, dim red light with a wavelength >620 nm was used, as this wavelength does not suppress melatonin production in rodents. All routine procedures (food change, bedding replacement, cage cleaning) were performed at standardized times (between 10:00 AM and 11:00 AM) for all groups [69,70].

The animals were randomly divided into three equal groups (*n* = 40 each):

1. Control group (LD): Fixed light schedule with a 10:14 h light:dark cycle (lights on at 8:00, lights off at 18:00).
2. Group I (constant light, LL): Continuous light exposure (24 h of light per day).
3. Group II (intermittent constant light/recovery regimen, ICL): A cyclically repeating regimen consisting of 1 day of constant light followed by 2 days of normal photoperiod (10:14 h LD). This 3-day cycle was repeated 7 times over the 21-day experimental period.

### 4.2. Study Design

Experimental timeline: 14-day acclimatization phase followed by a 21-day experimental phase. Animals were sacrificed on the 22nd day.

Tissue collection time points: Four time points across a 24 h cycle: 9:00 AM, 3:00 PM, 9:00 PM, 3:00 AM.

Sampling design: Cross-sectional—different animals at each time point (*n* = 10 per group per time point, total *n* = 40 per group).

Timing for Group ICL: Tissue collection was performed 48 h after the last transition from LL to LD (i.e., during the second day of the recovery LD phase).

Euthanasia procedure: Animals were anesthetized with diethyl ether vapor to deep anesthesia (loss of corneal reflex and pedal withdrawal reflex), followed by decapitation. Decapitation was chosen for rapid tissue collection for ultrastructural analysis without pharmacological agents that could affect blood biochemical parameters and tissue ultrastructure [71,72,73]. The time from cage removal to tissue collection was <2 min for each animal.

Sample collection:

- Blood: Collected from the trunk after decapitation into heparinized tubes. Plasma was separated by centrifugation at 3000 rpm for 15 min at 4 °C and stored at −80 °C.
- Pineal gland: Rapidly dissected (within 3 min of decapitation). The gland was divided in half: one half fixed in 10% neutral buffered formalin; the other half (2 mm^3^ samples) fixed in 2.5% glutaraldehyde for electron microscopy.

### 4.3. Morphological and Morphometric Methods

Half of the extracted pineal glands were fixed in 10% neutral buffered formalin, followed by passage through increasing concentrations of alcohol and xylenes, and embedding in Histomix histological medium (BioVitrum, St. Petersburg, Russia). Serial histological sections (3–4 µm thick) were prepared on a Leica SM2010 R rotary microtome (Leica Biosystems, Wetzlar, Germany). Hematoxylin and eosin staining was performed according to the standard technique.

Microscopy was performed on a Leica DM 2500 digital microscope with a Leica DFC 290 digital camera (Leica Biosystems, Germany).

Sampling scheme for light microscopy:

- Number of animals: *n* = 10 per group per time point;
- Number of sections per animal: 5–7 serial histological sections;
- Number of fields per section: 10 digital images at ×400 magnification;
- Cells analyzed per animal: 35–50 light pinealocytes, 20–30 dark pinealocytes.

Morphometric parameters measured: Using Fiji software (ImageJ v2), nuclear area (Sn, µm^2^), nuclear perimeter (Pn, µm), and cell profile area (Sc, µm^2^) were determined.

Statistical unit: Animal-level averages were used as biological replicates; individual cells/fields were treated as technical replicates.

### 4.4. Electron Microscopy

Pineal gland samples (2 mm^3^) were fixed with 2.5% glutaraldehyde in phosphate buffer (pH 7.4), postfixed in 1% osmium tetroxide, dehydrated in ethanol, counterstained with 1% uranyl acetate, and embedded in Epon-Araldite.

Ultrathin sections (~70 nm) obtained on a UC Enuity ultratome (Leica Microsystems CMS GmbH, Wetzlar, Germany) were counterstained with Reynolds lead citrate and examined under a HIMERA EM50X field emission scanning electron microscope (CIQUTEK, Shanghai, China) at 80 kV.

Sampling scheme for electron microscopy:

- Number of animals: *n* = 10 per group per time point;
- Number of sections per animal: 2–3 ultrathin sections;
- Number of fields per animal: ≥20 at ×20,000 magnification, 10 at ×50,000 magnification;
- Mitochondria analyzed per animal: 40–60 mitochondria;
- Cells analyzed per animal: 35–50 light pinealocytes, 20–30 dark pinealocytes.

Morphometric parameters measured: Using QuPath (QuPath-v0.7.0) and ImageJ, mitochondrial profile area and perimeter, number of cristae, mitochondrial fraction (%), Golgi complex fraction (%), lipid droplet area (µm^2^), and lysosome area (µm^2^).

Statistical unit: Animal-level averages were used as biological replicates; individual organelles/fields were treated as technical replicates [74].

### 4.5. Immunohistochemical Analysis

Immunohistochemical staining was performed automatically on an Autostainer BenchMark ULTRA (Ventana, Roche, Mannheim, Germany) using OptiView DAB Detection Kit (Roche, Germany).

Controls:

- Positive control: Rat liver sections;
- Negative controls: Isotype control (rabbit IgG), no primary antibody, pre-adsorption control.

Scoring: Semi-quantitative 0–3 scale (0: no staining; 1: weak; 2: moderate; 3: intense). H-score = Σ (% cells × score), ranging 0–300.

Sample size: *n* = 10 per group per time point.

Statistical unit: Animal-level average H-score.

Blinding: All analyses performed by two independent investigators blinded to group allocation (Cohen’s κ: PER2 = 0.84, CLOCK = 0.82).

### 4.6. Biochemical Analysis

Blood plasma biochemical parameters (total protein, albumin, glucose, cholesterol, triglycerides, LDH, alkaline phosphatase) were measured using an ACCENT S120 automatic analyzer (Cormay, Lomianki, Poland).

### 4.7. ELISA

Melatonin concentration in blood was determined by enzyme immunoassay using “ELISA Kit for Melatonin” (Cloud-Clone Corp., Wuhan, China) on an ADC ELISA 200 analyzer.

### 4.8. Measuring Rectal Temperature

Rectal temperature was measured using a BIO-TK8851 thermometer (Bioseb, Pinellas Park, FL, USA).

### 4.9. Blinding

Blinding was implemented at all analytical stages. Samples were assigned random identification numbers by an independent laboratory assistant. Investigators performing histological processing, electron microscopy, morphometric measurements, and biochemical/ELISA assays were blinded to group allocation.

### 4.10. Statistical Analysis

Statistical analysis was performed using GraphPad Prism v8.41 and R (version 4.4.1).

Biological replicates: The individual animal was the biological replicate. Animal-level averages were used for all analyses.

Sample sizes:

- Body weight, temperature, biochemistry, ELISA: *n* = 10 per group per time point;
- Morphometry (light and electron microscopy): *n* = 10 per group per time point;
- Immunohistochemistry: *n* = 5 per group per time point.

Nested data: Linear mixed models with “animal” as random effect; animal-averaged values were used when random effect was non-significant.

Normality: Shapiro–Wilk test (primary) and D’Agostino–Pearson test; Q-Q plots. For n < 8, non-parametric methods were used.

Univariate analysis:

- Two-factor ANOVA (Group × Time) with Tukey’s HSD post hoc;
- Non-parametric: Kruskal–Wallis with Dunn’s test.

Cosinor analysis: Y(t) = M + A · cos(2πt/τ + φ), τ = 24 h. Significance: 95% confidence ellipse not passing through the origin and F-test *p* < 0.05 [75,76,77]. Phase comparisons: Watson–Williams test.

Multiple comparisons: Benjamini–Hochberg FDR correction (q < 0.05).

Effect size: Hedges’ g (|g| ≥ 0.2 small, ≥0.5 medium, ≥0.8 large); partial η^2^ (η^2^ ≥ 0.01 small, ≥0.06 medium, ≥0.14 large) [78].

Multivariate analysis:

- PCA: z-score standardization (prcomp).
- PERMANOVA: Bray–Curtis distance, 9999 permutations (adonis2).
- Elastic net: nested 5-fold cross-validation for α and λ tuning; performance metrics: accuracy, balanced accuracy, AUC.

Correlation analysis: Spearman’s ρ with FDR correction; |ρ| ≥ 0.5 considered meaningful.

Reporting: Results as mean ± SD; exact *p*-values and test statistics with degrees of freedom provided.

## 5. Conclusions

This comprehensive morphofunctional study of the pineal gland of Wistar rats under various lighting regimens has established the following:

1. Constant lighting causes complete flattening of the daily rhythms of light pinealocytes: nuclear area decreases by almost 30%, nuclear perimeter by 17%, cell profile area by 32%, and the proportion of dark pinealocytes increases by 18%. The mitochondrial fraction increases 1.5-fold with a 33% reduction in the number of cristae; the Golgi complex is suppressed almost 2-fold; and lipid droplet area increases by 51.8%. The lysosomal fraction increases 2-fold. The melatonin rhythm is absent, and its maximum level is reduced by more than 3.5-fold; PER2 and CLOCK protein abundance increases 1.8-fold without daily dynamics.
2. In the control group (fixed light–dark schedule 10:14 h), light pinealocytes exhibit a clear daily rhythm with a maximum in the evening hours, exceeding the minimum by 2.1-fold for nuclear area, 1.6-fold for perimeter, and 1.5-fold for cell profile area; dark pinealocytes show no dynamics. The nighttime peak of melatonin is 4.5 times higher than the daytime minimum. All studied biochemical parameters and body temperature have significant circadian rhythms with acrophases characteristic of nocturnal rodents.
3. The intermittent constant light/recovery regimen (24 h of constant light → two days of normal schedule) leads to partial recovery: the nuclear area of light pinealocytes is 25% higher than under constant light but 11% lower than the control; cell profile area is close to the control, but the nighttime peak is absent. The lysosomal fraction remains 2.4-fold higher than the control; the number of cristae is 18% lower than the control. The melatonin rhythm is significant, with its amplitude increased by 30%; PER2 and CLOCK protein rhythms are preserved with shifted acrophases and are increased 2.9-fold and 1.8-fold, respectively. Two days of the normal regimen are insufficient for complete restoration of mitochondrial function and nuclear architecture.
4. Under constant lighting, the rhythms of total protein, LDH, and creatinine are lost; the rhythms of albumin, glucose, cholesterol, triglycerides, and alkaline phosphatase are preserved with shifted acrophases. Under the intermittent regimen, rhythms of all parameters except LDH and creatinine are preserved, with multiple increases in amplitudes: glucose—5-fold, cholesterol—4.3-fold, alkaline phosphatase—6-fold. The body temperature rhythm is significant only in the control; it is flattened in both experimental groups.
5. Quantitative analysis of effect size showed that the lighting regimen accounts for more than 70% of the variance in ultrastructural and metabolic parameters. Constant lighting more severely damages ultrastructure (1.8-fold reduction of the Golgi complex, 30% decrease in nuclear area, 33% decrease in cristae number), whereas the intermittent regimen causes more pronounced metabolic shifts (2.4-fold increase in lysosomes, 4.3-fold increase in cholesterol rhythm amplitude, 2.9-fold increase in PER2 immunoreactivity). The most sensitive markers of the direct damaging action of light are the state of the Golgi complex, nuclear area of light pinealocytes, and cristae number; markers of the desynchronizing effect of the intermittent regimen are the lysosomal fraction, cholesterol level, and PER2 immunoreactivity.
6. Constant lighting and the intermittent constant light/recovery regimen induce qualitatively different types of light stress: the former acts as a direct damaging factor with pineal degeneration and loss of rhythms, while the latter acts as a desynchronizing factor with preserved structure but shifted acrophases, clock protein hyperactivation, and pronounced metabolic disturbances. The intermittent regimen reflects fragmented exposure to nighttime lighting with recovery periods, characteristic of some forms of irregular shift schedules, but does not constitute a full model of shift work.

The obtained data deepen our understanding of the pathogenesis of diseases associated with circadian rhythm disruption and open prospects for the development of targeted approaches to the prevention and correction of the consequences of light pollution and shift work. The identified differential markers can be recommended for preclinical assessment of the efficacy of chronobiotics and lighting normalization regimens.

## Figures and Tables

**Table 1:** Proportion of light and dark pinealocytes in the rat pineal gland.

Group	Light Cells, %	Dark Cells, %
LD (*n* = 40)	70.80 ± 3.87	29.12 ± 3.85
LL (*n* = 40)	65.41 ± 6.16 **	34.55 ± 6.16 **
ICL (*n* = 40)	62.90 ± 6.14 ***	37.10 ± 6.14 ***

Note. Data are presented as mean ± SD; *n* = 40 per group per Hereinafter, in table: ** (*p* ≤ 0.01), *** (*p* ≤ 0.001)—in comparison with the animals of the control group.

**Table 2:** Micromorphometric parameters of light pinealocytes.

Group	Nuclear Area, µm^2^	Nuclear Perimeter, µm	Cell Profile Area, µm^2^
LD (*n* = 40)	30.38 ± 1.97	23.08 ± 3.14	76.17 ± 6.77
LL (*n* = 40)	21.43 ± 1.54 ***	19.09 ± 3.17 ***	51.65 ± 5.40 ***
ICL (*n* = 40)	27.04 ± 4.51 ***, +++	22.53 ± 2.11 ++	74.34 ± 5.41 +

Note. Data are presented as mean ± SD; *n* = 40 per group per Hereinafter, in table: *** (*p* ≤ 0.001)—in comparison with the animals of the control group; + (*p* ≤ 0.05), ++ (*p* ≤ 0.01), +++ (*p* ≤ 0.001)—in comparison between animals of experimental groups.

**Table 3:** Micromorphometric parameters of dark pinealocytes.

Group	Nuclear Area, µm^2^	Nuclear Perimeter, µm	Cell Profile Area, µm^2^
LD (*n* = 40)	24.90 ± 1.72	21.92 ± 7.68	73.64 ± 3.88
LL (*n* = 40)	20.75 ± 2.11 ***	18.96 ± 6.84	60.88 ± 2.55 ***
ICL (*n* = 40)	23.17 ± 3.81 ++	20.94 ± 3.61	72.12 ± 3.27 +++

Note. Data are presented as mean ± SD; n = 40 per group per Hereinafter, in table: *** (*p* ≤ 0.001)—in comparison with the animals of the control group; ++ (*p* ≤ 0.01), +++ (*p* ≤ 0.001)—in comparison between animals of experimental groups.

**Table 4:** Profile areas of organelles and inclusions in light pinealocytes.

Group	Mitochondrial Fraction, %	Golgi Complex Fraction, %	Lipid Droplet Area, µm^2^	Lysosome Area, µm^2^
LD (*n* = 40)	19.10 ± 1.27	2.31 ± 0.19	4.40 ± 0.37	0.30 ± 0.032
LL (*n* = 40)	28.15 ± 1.83 ***	1.25 ± 0.19 ***	2.12 ± 0.16 ***	0.59 ± 0.098 ***
ICL (*n* = 40)	27.16 ± 2.44 ***, +++	1.72 ± 0.20 ***, +++	2.00 ± 0.15 ***, ++	0.72 ± 0.062 ***, +++

Note. Data are presented as mean ± SD; *n* = 40 per group per Hereinafter, in table: *** (*p* ≤ 0.001)—in comparison with the animals of the control group; ++ (*p* ≤ 0.01), +++ (*p* ≤ 0.001)—in comparison between animals of experimental groups.

**Table 5:** Micromorphometric parameters of mitochondria in light pinealocytes.

Group	Mitochondrial Profile Area, µm^2^	Mitochondrial Perimeter, µm	Number of Cristae
LD (*n* = 40)	0.36 ± 0.057	2.13 ± 0.11	15.01 ± 0.93
LL (*n* = 40)	0.22 ± 0.077 ***	1.71 ± 0.16 ***	10.06 ± 1.43 ***
ICL (*n* = 40)	0.23 ± 0.08 ***, +++	1.75 ± 0.16 ***, +++	11.90 ± 2.20 ***, +++

Note. Data are presented as mean ± SD; *n* = 40 per group per Hereinafter, in tables: *** (*p* ≤ 0.001)—in comparison with the animals of the control group; +++ (*p* ≤ 0.001)—in comparison between animals of experimental groups.

**Table 6:** Average daily concentrations of biochemical parameters in rat blood.

**Parameter**	LD (*n* = 40)	LL (*n* = 40)	ICL (*n* = 40)
**Total protein**, g/L	98.25 ± 17.38	80.19 ± 15.94 ***	88.97 ± 13.39 *
**Albumin**, g/L	61.04 ± 4.61	18.25 ± 3.29 ***	25.58 ± 5.85 ***, +++
**Cholesterol**, mmol/L	2.01 ± 0.12	2.45 ± 0.21 ***	2.68 ± 0.30 ***, ++
**Triglycerides**, mmol/L	0.91 ± 0.08	1.22 ± 0.11 ***	1.35 ± 0.16 ***, ++
**LDH**, U/L	489.8 ± 91.7	769.3 ± 302.8 **	584.0 ± 253.2
**Alkaline phosphatase**, U/L	356.3 ± 64.21	340.8 ± 50.66	566.0 ± 356.8 *
**Glucose**, mmol/L	5.14 ± 1.24	6.65 ± 1.44 **	5.15 ± 2.1 +

Note. Data are presented as mean ± SD; *n* = 40 per group per Hereinafter, in table: * (*p* ≤ 0.05), ** (*p* ≤ 0.01), *** (*p* ≤ 0.001)—in comparison with the animals of the control group; + (*p* ≤ 0.05), ++ (*p* ≤ 0.01), +++ (*p* ≤ 0.001)—in comparison between animals of experimental groups.

**Table 7:** Results of cosinor analysis of the daily dynamics of biochemical parameters.

Parameter	LD (n = 40 (10))	LL	ICL
**Total protein**	Acrophase 6:42, A = 5.71	Rhythm absent	Acrophase 12:28, A = 13.47
**Albumin**	Acrophase 9:02, A = 2.83	Acrophase 21:44, A = 2.87	Acrophase 15:08, A = 5.10
**Glucose**	Acrophase 6:42, A = 0.39	Acrophase 7:37, A = 0.67	Acrophase 10:19, A = 1.99
**Cholesterol**	Acrophase 11:56, A = 0.21	Acrophase 11:57, A = 0.66	Acrophase 14:02, A = 0.90
**Triglycerides**	Acrophase 2:22, A = 0.092	Acrophase 13:42, A = 0.076	Acrophase 19:51, A = 0.099
**LDH**	Acrophase 5:30, A = 35.71	Rhythm absent	Rhythm absent
**Alkaline phosphatase**	Acrophase 5:24, A = 61.38	Acrophase 14:08, A = 67.66	Acrophase 3:10, A = 372.65

Note: A—amplitude (in the units of measurement of the parameter). Rhythm considered significant when the 95% confidence ellipse does not pass through the origin and F-test *p* < 0.05. *n* = 40 (10 per group per time point).

**Table 8:** Most sensitive parameters by effect size (η^2^).

Parameter	η^2^	Interpretation
**CLOCK immunoreactivity**	0.963	>95% of variance explained by light
**PER2 immunoreactivity**	0.954	>95% of variance explained by light
**Lipid droplet fraction**	0.954	>95% of variance explained by light
**Lysosome fraction**	0.910	>90% of variance explained by light
**Golgi complex fraction**	0.839	>80% of variance explained by light
**Mitochondrial fraction**	0.834	>80% of variance explained by light
**Light cell profile area**	0.772	>75% of variance explained by light

## Data Availability

The data underlying this article will be deposited in the Zenodo repository upon acceptance of the manuscript.
